# Site-specific associations between miRNA expression and survival in colorectal cancer cases

**DOI:** 10.18632/oncotarget.11173

**Published:** 2016-08-10

**Authors:** Martha L. Slattery, Jennifer S. Herrick, Daniel F. Pellatt, Lila E. Mullany, John R. Stevens, Erica Wolff, Michael D. Hoffman, Roger K. Wolff, Wade Samowitz

**Affiliations:** ^1^ Department of Medicine, University of Utah, Salt Lake City, Utah 84108, USA; ^2^ Department of Mathematics and Statistics, Utah State University, Logan, Utah 84322, USA; ^3^ Department of Pathology, University of Utah School, Salt Lake City, Utah 84108, USA

**Keywords:** colon cancer, rectal cancer, miRNA, disease stage

## Abstract

**Background:**

MicroRNAs (miRNA) are small non-coding RNA involved in cellular processes, including cell proliferation and angiogenesis. Thus, miRNA expression may alter survival after diagnosis with colorectal cancer (CRC).

**Results:**

Individuals diagnosed with stage 1 or stage 2 rectal cancer had worse survival than colon cancer cases diagnosed at stage 1 or stage 2. After adjustment for multiple comparisons, no miRNAs were significantly associated with disease stage. Two miRNAs infrequently expressed in the population and not previously reported were associated with survival after diagnosis with colon cancer (miR-1 HR 2.17 95% CI 1.41, 3.36; and miR-101-3p HR 3.51 95% CI 1.72, 7.15). Among those diagnosed with rectal cancer, 201 miRNAs were associated with survival when the FDR *q* value was < 0.05. Assessment of 105 previously reported miRNAs associated with prognosis showed that four miRNAs influenced colon cancer survival and 17 influenced survival after a diagnosis with rectal cancer when raw *p* values were considered.

**Patients and Methods:**

This study includes data from population-based studies of CRC conducted in Utah and the Kaiser Permanente Medical Care Program. A total of 1893 carcinoma and normal paired colorectal mucosa tissue samples were run using the Agilent Human miRNA Microarray V19.0. We assessed miRNA differential expression between paired carcinoma and normal colonic mucosa tissue with CRC- specific survival evaluating stage and site-specific associations after adjusting for age, sex, microsatellite instability tumor status, and AJCC stage.

**Conclusions:**

MiRNAs dysregulated for both colon and rectal cancer had a greater impact on survival after a diagnosis with rectal cancer.

## INTRODUCTION

MicroRNAs (miRNA) are small non-coding RNAs that regulate gene expression and are thus involved in numerous physiological and cellular processes, including tumor initiation and growth, cell proliferation, apoptosis, and angiogenesis [1–3]. We have shown that miRNAs are extensively dysregulated in colorectal carcinoma (CRC) [4]. Given the extensive role of miRNAs in gene regulation and cellular processes, the evaluation of miRNAs as regulators of tumor aggressiveness and prognosis is of interest [5, 6]. To this end, several miRNAs have been shown to be associated with either disease stage or survival after diagnosis with CRC [7]. Most studies have focused on select miRNAs, such as miR-21, in sample sets that are relatively small [8–10]. Our earlier attempt to replicate these findings in a sample of 1141 cases of CRC looking at miRNA expression in tumors showed that few of these candidate miRNAs replicated in terms of survival [11].

The importance of evaluating prognostic effects for colon and rectal cancer separately may provide clues to site-specific important miRNAs in terms of survival. We have previously shown that tumors with microsatellite instability (MSI) behavior differently in terms of survival for colon and rectal cancers, with colon tumors having better survival and rectal tumors having worse survival when they are unstable [12, 13]. Additionally we have shown that unique dysregulation of miRNAs for MSI vs microsatellite stable (MSS) cancers is more pronounced than for any other tumor phenotype (tumor phenotype paper in press) [14].

In this study, we examine site-specific associations with colon and rectal cancer with survival. We demonstrate that low stage rectal carcinomas have significantly poorer survival than low stage colon carcinomas. We evaluate the associations with survival between miRNAs and disease stage within colon and rectal carcinomas separately. We utilize a large set of 1134 colon carcinomas and 721 rectal carcinomas to determine if differential expression between carcinomas and normal colorectal mucosa influence survival. We utilize these data to implement both a discovery stage examining miRNAs from an Agilent platform that have not been previously associated with survival as well as evaluate 105 miRNAs that have been associated in the literature with either stage or survival in order to replicate previous findings.

## RESULTS

**Figure 1:**
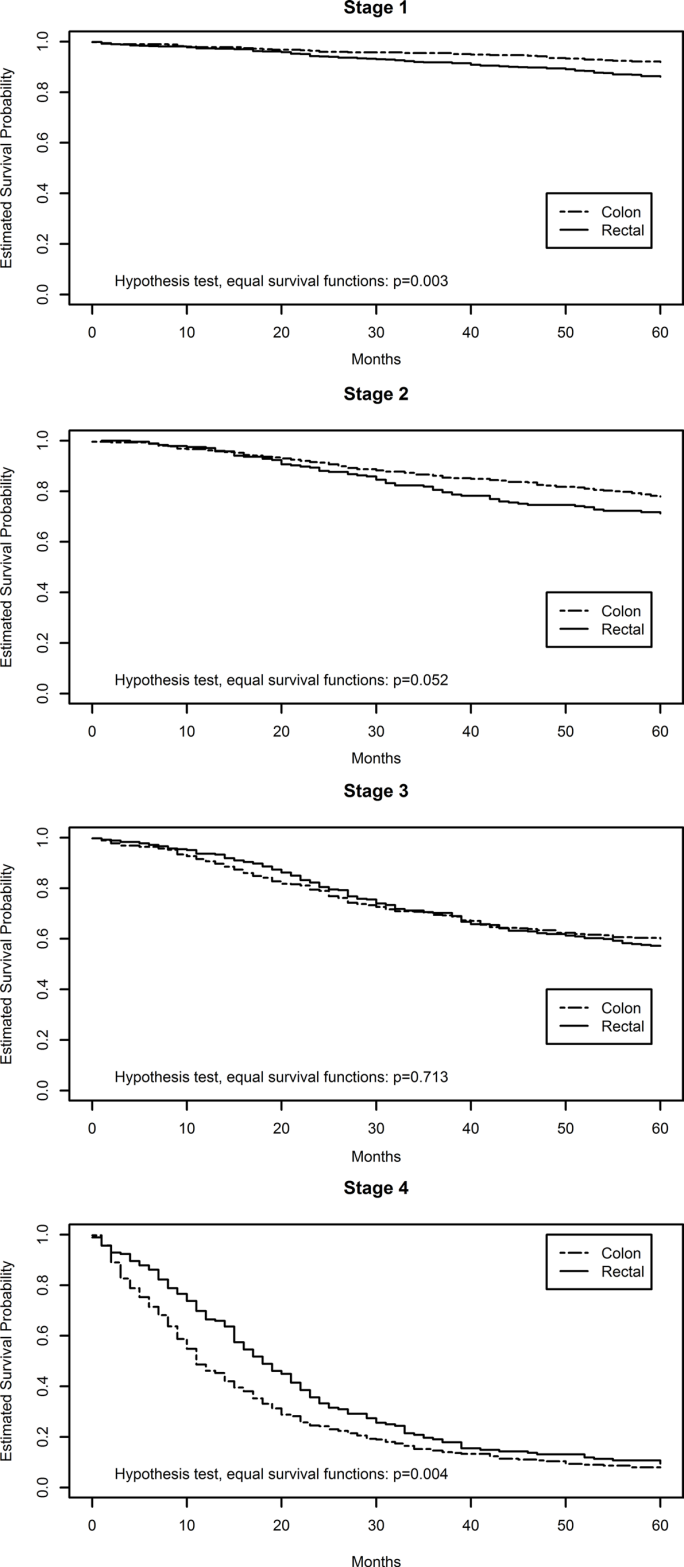
Kaplan meier plots of colon and rectal cancer survival by disease stage

Slightly more than half of the study subject were male (Table 1). Approximately half of the colon carcinomas were located in the proximal colon and half were located in the distal colon. Slightly less than half of the study participants had died at the time of last follow-up; the average follow-up time was 60.4 months. Figure 1 shows five-year survival after diagnosis with colon or rectal cancer by stage at time of diagnosis. The *p*-values in this figure are those associated with the Mantel-Haenszel/log-rank test for equal survival functions. Rectal cancers diagnosed at either stage 1 or 2 had significantly poorer survival than those diagnosed with stage 1 and 2 colon cancer, however for both colon and rectal cancer diagnosed at stage 1 over 90% survived while over 80% survived for both colon and rectal cancers diagnosed at stage 2. However, stage 4 rectal cancers had better survival than stage 4 colon cancers.

**Table 1:** Description of study population

	Overall		Colon		Rectal	
	*N*	%	*N*	%	*N*	%
Sex						
Male	1003	54.1	597	52.6	406	56.3
Female	852	45.9	537	47.4	315	43.7
Center						
Kaiser	1128	60.8	726	64.0	402	55.8
Utah	727	39.2	408	36.0	319	44.2
Site						
Proximal	559	49.4	559	49.3	0	0.0
Distal	573	50.6	573	50.5	0	0.0
Study						
Stage I	556	30.0	257	22.7	299	41.5
Stage II	487	26.3	348	30.7	139	19.3
Stage III	546	29.4	339	29.9	207	28.7
Stage IV	266	14.3	190	16.8	76	10.5
Vital Status						
Dead	892	48.1	554	48.9	338	46.9
Alive	963	51.9	580	51.1	383	53.1
	Mean	STD	Mean	STD	Mean	STD
Survival Months	60.4	33.8	60.4	35.4	60.3	31.1
Age	64.2	10.2	65.4	9.5	62.3	11.0

### Stage-specific analysis

We observed no stage-specific associations of miRNA's with survival after adjustment for multiple comparisons for either colon or rectal carcinomas (Supplementary Tables S1 for colon and Supplementary Tables S2 for rectal show those with *p* values of < 0.05 prior to adjustment for multiple comparisons). Prior to adjustment for multiple comparisons seven miRNAs were associated with survival for stages 1 and 2 for colon cancer and 17 miRNAs were associated with survival for more advanced carcinomas. For rectal cancer, 44 differentially expressed miRNAs were associated with survival for stage 1 and 2 carcinomas and 150 were associated with survival for carcinomas at more advanced stages. Of the miRNAs associated with survival by stage for rectal cancer, 13 were associated similarly for stages 1 and 2 and for stages 3 and 4. Two of the miRNAs associated with survival for stages 1 and 2, miR-429 and miR-4461, were associated similarly with survival for both colon and rectal carcinoma.

In an attempt to identify miRNAs that could uniquely contribute to survival after diagnosis with rectal cancer in stages 1 or 2, we compared the miRNAs associated with survival for stage 1 and 2 rectal cancer with those associated with survival for colon stage 1 and 2, rectal stage 3 and 4, and stage-adjusted rectal carcinoma. In this exploratory analysis, we observed that 20 miRNAs that were uniquely associated with survival for stages 1 and 2 rectal carcinomas (Table 2).

**Table 2:** MicroRNA associated with survival in stage 1 and 2 rectal cancer

	miRNA	% expressing	Q1	Q3	HR^1^	95% (CI)	*p*-value	FDR *q*-value
Associated in Both Colon and Rectal Stage 1 and 2	hsa-miR-429	65.9	0.00	2.06	0.77	(0.62, 0.96)	0.032	0.294
	hsa-miR-4461	65.7	–0.35	0.79	0.81	(0.68, 0.98)	0.031	0.294
Associated In Rectal Stage 1 & 2 and in Stage 3 & 4	hsa-miR-1228-5p	96.6	−0.17	0.41	0.82	(0.71, 0.96)	0.017	0.294
	hsa-miR-129-5p	98.6	−0.44	0.00	1.28	(1.05, 1.57)	0.017	0.294
	hsa-miR-130a-3p	52.4	−0.61	1.60	0.74	(0.57, 0.97)	0.043	0.294
	hsa-miR-30e-5p	46.8	−1.51	0.39	0.71	(0.52, 0.97)	0.044	0.294
	hsa-miR-3188	100.0	−0.56	−0.06	1.31	(1.02, 1.68)	0.045	0.294
	hsa-miR-3679-5p	100.0	−0.49	0.02	1.29	(1.01, 1.65)	0.049	0.294
	hsa-miR-4461	65.7	−0.35	0.79	0.81	(0.68, 0.98)	0.031	0.294
	hsa-miR-4470	100.0	−0.41	−0.08	1.32	(1.05, 1.65)	0.021	0.294
	hsa-miR-4505	100.0	−0.46	0.03	1.32	(1.05, 1.65)	0.025	0.294
	hsa-miR-4687-3p	100.0	−0.60	−0.05	1.33	(1.04, 1.70)	0.035	0.294
	hsa-miR-6124	100.0	−0.34	0.19	1.32	(1.05, 1.67)	0.025	0.294
	hsa-miR-6125	100.0	−0.65	−0.10	1.30	(1.02, 1.66)	0.047	0.294
	hsa-miR-6722-3p	100.0	−0.44	0.05	1.31	(1.02, 1.68)	0.048	0.294
Associated in only Stage 1 and 2 rectal and not any colon or rectal other stage or overall	hsa-miR-139-3p	99.6	−0.57	−0.20	1.31	(1.05, 1.64)	0.021	0.294
	hsa-miR-26a-5p	99.2	−0.34	0.63	0.76	(0.58, 0.99)	0.041	0.294
	hsa-miR-3185	99.9	−0.30	0.16	1.29	(1.02, 1.63)	0.048	0.294
	hsa-miR-3187-3p	97.7	−0.32	0.15	1.21	(1.04, 1.40)	0.018	0.294
	hsa-miR-3666	98.9	−0.35	0.04	1.29	(1.04, 1.60)	0.023	0.294
	hsa-miR-422a	98.4	−0.26	0.11	1.19	(1.02, 1.38)	0.025	0.294
	hsa-miR-4299	100.0	−0.51	−0.01	1.29	(1.05, 1.60)	0.022	0.294
	hsa-miR-4323	97.9	−1.01	−0.36	1.29	(1.03, 1.61)	0.025	0.294
	hsa-miR-4450	65.6	−0.16	0.85	0.84	(0.71, 0.98)	0.033	0.294
	hsa-miR-4507	100.0	−0.50	0.01	1.28	(1.02, 1.60)	0.050	0.294
	hsa-miR-4660	70.3	−0.08	0.76	0.87	(0.76, 0.99)	0.039	0.294
	hsa-miR-4725-3p	97.4	−0.41	0.04	1.25	(1.01, 1.54)	0.028	0.294
	hsa-miR-4749-3p	95.9	−0.95	−0.34	1.36	(1.08, 1.70)	0.006	0.294
	hsa-miR-4787-5p	100.0	−0.74	−0.13	1.34	(1.05, 1.72)	0.029	0.294
	hsa-miR-489	96.9	−0.30	0.11	1.17	(1.04, 1.31)	0.020	0.294
	hsa-miR-5001-5p	100.0	−0.66	−0.07	1.30	(1.02, 1.65)	0.050	0.294
	hsa-miR-5010-5p	97.4	−0.17	0.18	0.89	(0.81, 0.97)	0.013	0.294
	hsa-miR-508-5p	80.4	−0.43	0.12	0.83	(0.74, 0.94)	0.007	0.294
	hsa-miR-5195-5p	85.9	−0.13	0.60	0.86	(0.77, 0.95)	0.006	0.294
	hsa-miR-548q	100.0	−0.79	−0.17	1.38	(1.07, 1.78)	0.017	0.294
	hsa-miR-6074	99.3	−0.16	0.14	1.18	(1.04, 1.33)	0.018	0.294
	hsa-miR-650	77.7	−2.67	−1.22	0.79	(0.60, 1.03)	0.046	0.294
	hsa-miR-654-5p	99.8	−0.68	−0.18	1.34	(1.06, 1.70)	0.018	0.294

1 Hazard Ratios (HR) and 95% Confidence Intervals (CI) adjusted for age, sex, and MSI status and reflect the difference between the 75th and 25th percentile. HR reflect survival risk among those with stage 1 and 2 rectal cancer.

### miRNAs in discovery component

In the discovery component of the study, examination of miRNAs commonly expressed (i.e. those expressed in at least 50% of the population) showed no significant findings for survival and colon cancer after adjustment for multiple comparisons for either site overall or any specific site within the colon; we did not see a significant interaction between miRNAs and mortality for the progression of colon tumor site from cecum to sigmoid colon. However, 19 miRNAs were associated with survival prior to adjustment for multiple comparisons (Supplementary Table S3). On the other hand, for rectal cancer we observed that differential expression of 201 miRNAs were associated with survival when the FDR *q* value was < 0.05 (Table 3 for those with *q* value of < 0.031 and Supplementary Table S4 for those with *q* value between 0.031 and < 0.05); 228 miRNAs were associated with survival after diagnosis with rectal cancer prior to adjustment for multiple comparisons. The majority of HR were modest with the strongest associations for the interquartile range of differential expression being miR- 30e-5p (HR 0.69 95% CI 0.56, 0.84) for tumors having up-regulated expression of this miRNA and for miR-6131 for increased risk when this miRNA was not down-regulated in tumors (HR 1.31 95% CI 1.12, 1.54). Five canonical pathways were significantly associated with those miRNAs that reduced risk of dying after adjustment for multiple comparisons. Those pathways were integrin signaling, actin cytoskeleton signaling, epithelial adherens junction signaling, ILK signaling, and ERK/MAPK signaling.

**Table 3:** Differential miRNA expression between carcinoma and normal mucosa associated with survival in rectal cancer cases where FDR q value is < 0.031

miRNA	% expressing	25th%ile	75th%ile	HR^1^	95% (CI)	*p*-value	FDR *q*-value
hsa-miR-1182	99.9	−0.11	0.23	1.25	(1.10, 1.42)	0.0013	0.0231
hsa-miR-1183	100.0	−0.17	0.12	1.18	(1.05, 1.34)	0.0087	0.0305
hsa-miR-1207-5p	100.0	−0.51	0.01	1.24	(1.07, 1.44)	0.0055	0.0278
hsa-miR-1225-5p	100.0	−0.49	0.00	1.24	(1.07, 1.45)	0.0081	0.0304
hsa-miR-1228-5p	95.0	−0.16	0.41	0.84	(0.77, 0.93)	0.0009	0.0231
hsa-miR-1229-5p	100.0	−0.45	0.08	1.25	(1.09, 1.44)	0.0024	0.0247
hsa-miR-1233-1-5p	100.0	−0.40	–0.03	1.30	(1.12, 1.50)	0.0007	0.0231
hsa-miR-1234-5p	100.0	−0.55	–0.04	1.23	(1.06, 1.42)	0.0096	0.0305
hsa-miR-1288	100.0	−0.56	0.00	1.30	(1.11, 1.53)	0.0012	0.0231
hsa-miR-129-5p	99.0	−0.43	0.01	1.21	(1.09, 1.35)	0.0010	0.0231
hsa-miR-1305	100.0	−0.57	–0.03	1.24	(1.07, 1.45)	0.0063	0.0283
hsa-miR-130a-3p	54.8	−0.86	1.55	0.75	(0.62, 0.90)	0.0018	0.0242
hsa-miR-139-3p	100.0	−0.57	–0.19	1.20	(1.05, 1.37)	0.0088	0.0305
hsa-miR-142-3p	57.5	−1.51	0.00	0.82	(0.71, 0.94)	0.0027	0.0247
hsa-miR-146a-5p	73.5	−0.51	1.46	0.78	(0.67, 0.90)	0.0007	0.0231
hsa-miR-15a-5p	62.9	−0.13	1.69	0.77	(0.66, 0.89)	0.0011	0.0231
hsa-miR-1915-3p	100.0	−0.62	–0.06	1.27	(1.09, 1.47)	0.0036	0.0247
hsa-miR-194-5p	99.6	−1.06	0.27	0.83	(0.72, 0.94)	0.0098	0.0305
hsa-miR-196b-5p	71.0	0.00	3.20	0.75	(0.61, 0.93)	0.0093	0.0305
hsa-miR-198	100.0	−0.31	0.08	1.28	(1.09, 1.50)	0.0029	0.0247
hsa-miR-19b-3p	83.8	0.81	2.39	0.80	(0.70, 0.92)	0.0015	0.0231
hsa-miR-202-3p	99.9	−0.29	0.06	1.23	(1.08, 1.40)	0.0035	0.0247
hsa-miR-2392	100.0	−0.33	0.11	1.19	(1.06, 1.35)	0.0060	0.0282
hsa-miR-30c-1-3p	100.0	−0.39	0.00	1.30	(1.11, 1.53)	0.0017	0.0239
hsa-miR-30e-5p	65.7	−1.57	0.40	0.69	(0.56, 0.84)	0.0003	0.0231
hsa-miR-3125	100.0	−0.41	0.05	1.24	(1.07, 1.44)	0.0055	0.0278
hsa-miR-3127-5p	100.0	−0.43	–0.01	1.22	(1.07, 1.41)	0.0040	0.0247
hsa-miR-3187-3p	98.9	−0.29	0.15	1.17	(1.06, 1.31)	0.0033	0.0247
hsa-miR-3188	100.0	−0.54	–0.06	1.26	(1.09, 1.45)	0.0042	0.0247
hsa-miR-3195	100.0	−0.62	–0.03	1.26	(1.07, 1.47)	0.0074	0.0299
hsa-miR-3198	100.0	−0.37	0.13	1.30	(1.11, 1.52)	0.0015	0.0231
hsa-miR-3609	51.2	−0.33	0.88	0.83	(0.73, 0.94)	0.0030	0.0247
hsa-miR-3621	100.0	−0.37	0.04	1.25	(1.09, 1.44)	0.0033	0.0247
hsa-miR-3653	98.2	−0.74	0.13	0.85	(0.75, 0.96)	0.0085	0.0305
hsa-miR-3667-5p	99.9	−0.34	0.12	1.22	(1.07, 1.38)	0.0031	0.0247
hsa-miR-3679-5p	100.0	−0.47	0.04	1.25	(1.08, 1.44)	0.0033	0.0247
hsa-miR-378b	100.0	−0.13	0.16	1.23	(1.08, 1.40)	0.0020	0.0247
hsa-miR-3945	100.0	−0.44	–0.02	1.24	(1.06, 1.45)	0.0081	0.0304
hsa-miR-3960	100.0	−0.51	0.11	1.28	(1.09, 1.50)	0.0040	0.0247
hsa-miR-422a	99.3	−0.25	0.12	1.12	(1.03, 1.22)	0.0056	0.0278
hsa-miR-4253	100.0	−0.27	0.08	1.28	(1.11, 1.48)	0.0013	0.0231
hsa-miR-425-3p	99.0	−0.55	0.09	1.17	(1.03, 1.32)	0.0088	0.0305
hsa-miR-4281	100.0	−0.45	0.10	1.25	(1.07, 1.45)	0.0061	0.0282
hsa-miR-429	65.4	−0.16	2.02	0.81	(0.70, 0.94)	0.0089	0.0305
hsa-miR-4294	100.0	−0.21	0.11	1.28	(1.11, 1.48)	0.0012	0.0231
hsa-miR-4299	100.0	−0.51	–0.02	1.20	(1.06, 1.35)	0.0043	0.0248
hsa-miR-431-5p	99.8	−0.64	–0.04	1.22	(1.09, 1.37)	0.0015	0.0231
hsa-miR-4443	100.0	−0.56	0.12	1.27	(1.09, 1.48)	0.0042	0.0247
hsa-miR-4461	71.4	−0.51	0.67	0.86	(0.77, 0.95)	0.0052	0.0278
hsa-miR-4470	100.0	−0.41	–0.08	1.26	(1.10, 1.45)	0.0014	0.0231
hsa-miR-4472	96.2	−0.49	0.10	1.13	(1.03, 1.24)	0.0098	0.0305
hsa-miR-4499	100.0	−0.51	0.00	1.28	(1.09, 1.50)	0.0028	0.0247
hsa-miR-4505	100.0	−0.44	0.05	1.24	(1.07, 1.42)	0.0040	0.0247
hsa-miR-4507	100.0	−0.49	0.03	1.21	(1.05, 1.39)	0.0094	0.0305
hsa-miR-4514	100.0	−0.24	0.11	1.20	(1.05, 1.37)	0.0081	0.0304
hsa-miR-4516	100.0	−0.59	–0.06	1.24	(1.06, 1.44)	0.0095	0.0305
hsa-miR-4534	100.0	−0.49	–0.01	1.24	(1.08, 1.42)	0.0037	0.0247
hsa-miR-4634	100.0	−0.58	0.07	1.27	(1.08, 1.49)	0.0070	0.0294
hsa-miR-4657	56.2	0.00	1.46	0.79	(0.67, 0.93)	0.0039	0.0247
hsa-miR-4673	100.0	−0.47	–0.07	1.21	(1.05, 1.39)	0.0071	0.0294
hsa-miR-4687-3p	100.0	−0.57	–0.04	1.28	(1.11, 1.49)	0.0013	0.0231
hsa-miR-4707-3p	90.4	−0.92	–0.07	1.18	(1.06, 1.31)	0.0069	0.0294
hsa-miR-4713-3p	100.0	−0.55	0.00	1.27	(1.07, 1.49)	0.0056	0.0278
hsa-miR-4716-3p	100.0	−0.51	–0.01	1.27	(1.09, 1.48)	0.0026	0.0247
hsa-miR-4730	56.8	−1.27	0.38	0.83	(0.73, 0.94)	0.0042	0.0247
hsa-miR-4739	100.0	−0.55	–0.02	1.25	(1.07, 1.46)	0.0093	0.0305
hsa-miR-4784	99.9	−0.24	0.04	1.17	(1.05, 1.31)	0.0061	0.0282
hsa-miR-4787-5p	100.0	−0.73	–0.12	1.24	(1.07, 1.44)	0.0063	0.0283
hsa-miR-4793-3p	77.8	−0.48	0.48	0.89	(0.82, 0.97)	0.0098	0.0305
hsa-miR-486-5p	100.0	−0.62	–0.17	1.23	(1.07, 1.43)	0.0059	0.0282
hsa-miR-5001-5p	100.0	−0.64	–0.04	1.23	(1.06, 1.44)	0.0087	0.0305
hsa-miR-508-5p	85.3	−0.38	0.13	0.91	(0.84, 0.97)	0.0085	0.0305
hsa-miR-550a-3-5p	100.0	−0.20	0.13	1.26	(1.08, 1.47)	0.0033	0.0247
hsa-miR-550b-2-5p	100.0	−0.37	0.01	1.23	(1.07, 1.42)	0.0073	0.0299
hsa-miR-5581-5p	100.0	−0.55	–0.03	1.26	(1.07, 1.49)	0.0050	0.0274
hsa-miR-572	100.0	−0.66	–0.10	1.27	(1.10, 1.47)	0.0032	0.0247
hsa-miR-6068	100.0	−0.65	–0.09	1.24	(1.07, 1.44)	0.0076	0.0300
hsa-miR-6087	100.0	−0.51	–0.05	1.24	(1.07, 1.42)	0.0059	0.0282
hsa-miR-6088	100.0	−0.47	0.06	1.28	(1.10, 1.48)	0.0017	0.0239
hsa-miR-6089	100.0	−0.58	–0.02	1.26	(1.08, 1.48)	0.0039	0.0247
hsa-miR-6090	100.0	−0.58	0.05	1.28	(1.09, 1.50)	0.0033	0.0247
hsa-miR-6124	100.0	−0.31	0.22	1.27	(1.10, 1.46)	0.0019	0.0246
hsa-miR-6125	100.0	−0.64	–0.08	1.26	(1.08, 1.47)	0.0040	0.0247
hsa-miR-6126	100.0	−0.56	–0.11	1.21	(1.06, 1.39)	0.0071	0.0294
hsa-miR-6131	100.0	−0.56	–0.03	1.31	(1.12, 1.54)	0.0010	0.0231
hsa-miR-623	100.0	−0.36	0.04	1.20	(1.06, 1.37)	0.0079	0.0304
hsa-miR-636	99.7	−0.57	0.09	1.24	(1.06, 1.45)	0.0045	0.0255
hsa-miR-638	100.0	−0.59	–0.07	1.24	(1.07, 1.44)	0.0083	0.0305
hsa-miR-654-5p	100.0	−0.69	–0.16	1.25	(1.08, 1.45)	0.0047	0.0262
hsa-miR-662	99.9	−0.34	0.02	1.18	(1.07, 1.30)	0.0033	0.0247
hsa-miR-671-5p	100.0	−0.44	0.05	1.19	(1.03, 1.37)	0.0095	0.0305
hsa-miR-6717-5p	100.0	−0.54	0.00	1.31	(1.12, 1.53)	0.0005	0.0231
hsa-miR-6722-3p	100.0	−0.39	0.06	1.30	(1.13, 1.49)	0.0007	0.0231
hsa-miR-6724-5p	100.0	−0.38	0.06	1.22	(1.06, 1.39)	0.0065	0.0288
hsa-miR-769-3p	100.0	−0.51	–0.06	1.26	(1.07, 1.49)	0.0068	0.0294
hsa-miR-874	100.0	−0.42	–0.05	1.21	(1.06, 1.39)	0.0076	0.0300
hsa-miR-877-5p	100.0	−0.22	0.13	1.22	(1.05, 1.41)	0.0096	0.0305
hsa-miR-892b	99.7	−0.62	–0.01	1.24	(1.07, 1.44)	0.0055	0.0278
hsa-miR-939-5p	100.0	−0.43	0.02	1.23	(1.08, 1.41)	0.0031	0.0247

1 Hazard Ratios (HR) and 95% Confidence Intervals (CI) adjusted for age, sex, AJCC stage, and MSI tumor status. HR are for interquartile range.

Evaluation of the 646 miRNAs that were expressed in less than 50% of the population, showed that any expression of two miRNAs, miR-1 and miR-101-3p, was associated with poorer survival after a diagnosis of colon cancer (Table 4). Having any expression of these miRNAs was associated with increased hazard of dying (HR 2.17 95% CI 1.41, 3.36 and HR 3.51 95% CI 1.72, 7.15 respectively). There were no survival-related associations for rectal cancer for miRNAs infrequently expressed.

**Table 4:** Hazard of dying after diagnosis with colon cancer associated with any miRNA expression in carcinoma tissue for miRNAs not commonly expressed

miRNA	% expressing	HR^1^	95% (CI)		*p* raw	FDR *q* value
hsa-miR-1	2.9	2.17	(1.41,	3.36)	0.00048	0.030
hsa-miR-101-3p	1.3	3.51	(1.72,	7.15)	0.00055	0.030

1 Hazard Ratios (HR) and 95% Confidence Intervals (CI) adjusted for age, sex, AJCC stage, and MSI tumor status.

### miRNAs in replication component

Assessment of replication of 52 previously reported miRNAs commonly expressed and associated with prognosis, showed that no miRNAs influenced colon cancer survival and 17 influenced survival after a diagnosis with rectal cancer (Table 5). These 17 miRNAs had a raw *p* value of < 0.05 and the miRNAs associated with rectal cancer had *q* values of < 0.1. Assessment of CRC-specific survival with less commonly expressed miRNAs showed two significant associations (*q* value < 0.05) with colon cancer survival and three associations, miR-224-5p, miR- 335-5p and miR- 374a-5p (*q* value < 0.1), with survival after being diagnosed with rectal carcinoma (Table 6).

**Table 5:** Hazard of dying associated with differential expression between carcinoma and normal mucosa of miRNAs previously reported in the literature

Study	miRNA	% expressing	25th%ile	75th%ile	HR^1^	95% (CI)	*p*-value	FDR *q*-value
Colon	hsa-miR-145-5p	100.0	–1.88	-0.04	1.14	(1.00, 1.31)	0.0442	0.998
	hsa-miR-148a-3p	81.6	–0.45	1.42	0.88	(0.79, 0.98)	0.0165	0.962
	hsa-miR-20b-5p	61.1	0.74	2.97	0.84	(0.74, 0.96)	0.0101	0.911
	hsa-miR-215	99.0	–1.53	-0.16	0.89	(0.82, 0.97)	0.0118	0.911
Rectal	hsa-miR-16-5p	99.3	–0.20	0.75	0.88	(0.78, 0.99)	0.0452	0.061
	hsa-miR-17-5p	95.9	1.11	2.43	0.80	(0.70, 0.92)	0.0030	0.025
	hsa-miR-20a-5p	94.6	1.19	2.69	0.80	(0.70, 0.92)	0.0034	0.025
	hsa-miR-20b-5p	61.1	1.19	3.27	0.68	(0.57, 0.80)	0.0001	0.023
	hsa-miR-215	99.0	–1.24	0.05	0.84	(0.74, 0.95)	0.0114	0.033
	hsa-miR-21-3p	87.0	0.50	1.62	0.86	(0.75, 0.97)	0.0237	0.040
	hsa-miR-221-3p	63.7	0.63	2.57	0.81	(0.70, 0.95)	0.0139	0.034
	hsa-miR-25-3p	89.8	0.63	1.83	0.88	(0.77, 0.99)	0.0429	0.060
	hsa-miR-29a-3p	99.1	0.49	1.74	0.79	(0.69, 0.90)	0.0010	0.023
	hsa-miR-29c-3p	90.0	–0.53	0.64	0.90	(0.82, 1.00)	0.0381	0.056
	hsa-miR-30c-5p	90.9	–0.92	0.18	0.89	(0.79, 1.00)	0.0451	0.061
	hsa-miR-34a-5p	91.2	0.24	1.44	0.86	(0.77, 0.97)	0.0124	0.033
	hsa-miR-370	100.0	–0.10	0.27	1.16	(1.02, 1.33)	0.0396	0.057
	hsa-miR-425-5p	80.3	0.00	1.54	0.85	(0.75, 0.96)	0.0116	0.033
	hsa-miR-490-5p	99.8	–0.35	0.01	1.13	(1.01, 1.26)	0.0354	0.053
	hsa-miR-500a-5p	99.8	–0.50	-0.02	1.30	(1.13, 1.51)	0.0009	0.023
	hsa-miR-92a-3p	99.6	0.78	1.86	0.81	(0.69, 0.95)	0.0114	0.033

1 Hazard Ratios (HR) and 95% Confidence Intervals (CI) adjusted for age, sex, AJCC stage, and MSI tumor status. HR are for interquartile range.

**Table 6:** Hazard of dying after diagnosis with colorectal cancer associated with any miRNA expression in carcinoma tissue for miRNAs not commonly expressed previously reported in the literature

Study	miRNA	% expressing	HR	95% (CI)	*p* value	FDR *q* value
Colon						
	hsa-miR-145-3p	1.9	3.36	(2.02, 5.59)	< 0.00001	0.0008
	hsa-miR-31-5p	17.2	1.70	(1.32, 2.19)	0.00004	0.0053
	hsa-miR-570-3p	2.5	0.33	(0.12, 0.89)	0.02859	0.3958
Rectal						
	hsa-miR-151a-3p	33.8	0.74	(0.57, 0.97)	0.02608	0.1013
	hsa-miR-224-5p	10.0	0.66	(0.49, 0.90)	0.00785	0.0875
	hsa-miR-335-5p	6.1	0.28	(0.13, 0.64)	0.00242	0.0875
	hsa-miR-374a-5p	23.0	0.47	(0.33, 0.68)	0.00006	0.0070

^1^Hazard Ratios (HR) and 95% Confidence Intervals (CI) adjusted for age, sex, AJCC stage, and MSI tumor status.

## DISCUSSION

Previously we reported that dysregulated miRNAs for colon and rectal carcinoma were almost identical [15]. Despite this, we observed that these dysregulated miRNAs had different effects on survival for colon and rectal carcinoma. While miRNAs had minimal impact on survival when dysregulated for colon cancer, the same miRNAs had considerable impact on survival after being diagnosed with rectal cancer. Some of the dysregulated miRNAs for rectal cancer appear to be uniquely associated with survival for stages 1 and 2 rectal cancer prior to adjustment for multiple comparisons. It is possible that these miRNAs may contribute to the decreased survival observed for stages 1 and 2 rectal cancer compared to stages 1 and 2 colon cancer. However, it should also be acknowledged that the FDR *q* value associated with these miRNAs was 0.29, thus, one would expect about 29% of associations to be null findings.

We have previously noted differences in survival between people diagnosed with colon or rectal cancer based on MSI status of tumors [12, 13]. MSI tumors were associated with worse survival for rectal tumors and better survival for colon tumors. Differences in survival after a diagnosis of colon or rectal cancer is also seen by AJCC disease stage in this study and supports previous reports [16]. We observed that people diagnosed with stage 1 and 2 rectal carcinomas had worse survival than those with stage 1 and 2 colon carcinomas. However people diagnosed with a stage 4 rectal tumor had slightly better survival than those with colon cancer diagnosed at stage 4, this is similar to observations from broader SEER data [16]. Although the differences in survival were statistically significant, there were few deaths for either colon or rectal cancer at stages 1 and 2. Over 90% of individuals diagnosed at AJCC Stage 1 survived 5 years for both colon and rectal cancer, while for AJCC Stage 2 the five year survival was over 80% for both tumor sites. However, as we observed, the differences between colon and rectal cancer were statistically significant and the reasons for these differences are not clear.

Perhaps one of the most important questions raised by this study is why the differences in survival associated with differential miRNA expression is seen for rectal cancer but not for colon cancer. This difference is even more puzzling when considering that the actual differential expression between carcinoma and normal coloreclt mucosa was almost identical [4, 17]. These differences in survival patterns cannot be explained by age differences, with rectal cancer cases being slightly younger than colon cancer cases. This would suggest that factors such as tumor microenvironment may play a major role in the ultimate prognosis associated with differentially expressed miRNAs. For instance it has been suggested that miRNAs act as modulators of angiogenesis [18] and that tumor microenvironment may further regulate angiogenesis [19]. Several of the miRNAs previously associated with angiogenesis, such as miR-17-5p, miR-20a, miR-221, miR-20, and miR-92 [18], were associated with survival after diagnosis with rectal cancer in our study. It is thus possible that the microenvironment of the rectum is sufficiently different than that of the colon cancer that once miRNAs are dysregulated, they have a greater impact on prognosis for rectal cancer than for colon cancer. Another possibility is the influence of preoperative chemo-radiation for rectal cancer may interact with the microenvironment or alter the microenvironment so that miRNA expression had a different impact on prognosis for rectal than colon carcinoma. Unfortunately, we are not able to test this hypothesis directly since treatment data at the time of diagnosis was not uniformly available.

In this study we incorporated both a discovery and a replication component. Because of our ability to evaluate 970 miRNAs expressed in colorectal tissue, we were able to assess site-specific associations with survival and disease stage for many miRNAs not previously assessed with prognosis. In this process we identified over a hundred miRNAs that could influence survival after diagnosed with rectal cancer. However, for the most part the impact on survival was not large, with most HRs for the interquartile range of differential expression being less than 1.3 after adjusting for age, AJCC disease stage, and MSI status.

In our previous study we reported replication of 121 miRNAs using 1141 of the colorectal samples that we included in this work [11]. In that replication we identified five miRNAs whose carcinoma expression levels were associated with advanced disease stage and 12 with colorectal cancer mortality among individuals diagnosed with colon cancer and 14 among individuals diagnosed with rectal cancer. In that work, we did not adjust for multiple comparisons since we were testing previously identified miRNAs and were therefore testing specific hypotheses. Additionally, we examined the level of miRNA expression in tumors in our previous analysis while here we examined differential expression and report both *p* values unadjusted for multiple comparisons as well as FDR *q* values. For those miRNAs previously identified with colon cancer and survival, we replicated results for four of the miRNA and nine for rectal cancer when looking at differential expression with survival using raw *p* values. However, with the larger sample and examination of differential expression in this current study, eight miRNAs that did not previously replicate with carcinoma miRNA expression were significantly associated with survival after diagnosis with rectal cancer. It is interesting to note that two of the miRNAs associated with survival after diagnosis with colon cancer and 12 of the miRNAs associated with survival after diagnosis with rectal cancer were still significant after adjustment for multiple comparisons with a *q* value of < 0.05 in our current study. Others who have evaluated miRNAs with survival and disease stage have not adjusted for multiple comparisons, which could account for some differences in significant associations.

The study has several strengths including the large sample size, the site-specific colorectal cancer data, data on MSI status, and AJCC stage data for all study participants. When assessing stage specific survival differences (Figure 1), we included data from all individuals diagnosed in the time period from the target geographic areas to have a population-based assessment of survival differences by stage and tumor site. The platform used enabled us to undertake both a discovery and replication study component. The Agilent platform has been shown to have excellent repeatability (*r* = 0.98) and relatively good agreement with Nanostring [4]. Comparison of Agilent results to qPCR showed 100% agreement in terms of directionality of dysregulation and almost 100% agreement in terms of the fold change associated with that dysregulation [17]. There are additionally several weaknesses we encountered that are applicable to any study of miRNAs. Bioinformatics tools to determine functionality of miRNAs associated with survival are very non-specific and incomplete. For instance, assessment of the 24 miRNAs associated uniquely with stages 1 and 2 rectal cancer and survival showed that they regulated thousands of validated genes. When focusing on the miRNAs associated with rectal cancer that were previously not reported as associated with survival, we identified 2942 target genes for those miRNAs associated with improved survival and 4224 target genes for those miRNAs associated with worse survival. Of those targeted genes, 2681 were targeted by more than one miRNA. Assessing those targeted genes with mRNA expression in 48 rectal samples, yielded 588 significant associations. Of those, 376 also had significant differential expression between carcinoma and normal rectal mucosa. Pathway analysis yielded five significant pathways associated with miRNAs that improved survival. Because of the number of miRNAs being examined, we adjusted for multiple comparisons; while this is a strength of the study, it is also a limitation of being able to relate findings to previously reported results. Most published studies have focused on a few miRNAs in small populations, making lack of confirmation of many previous findings not unexpected.

In conclusion, stage-specific prognosis differs for colon and rectal cancer; it is unclear the extent to which miRNAs contribute to this difference. We observed that miRNA differential expression in rectal carcinomas had a more pronounced impact on survival than they did for colon carcinomas. The majority of miRNAs identified as being associated with survival after diagnosis with rectal cancer were newly identified miRNAs not previously reported as being associated with survival. Replication of previous associations showed few remained significant in this large study, although differences in methodologies in both assessment of miRNAs and statistical methods used could contribute to these findings.

## MATERIALS AND METHODS

Study participants come from two population-based case-control studies that included all incident colon and rectal cancers between 30 and 79 years of age who resided along the Wasatch Front in Utah or were members of the Kaiser Permanente Medical Care Program (KPMCP) in Northern California. Participants were white, Hispanic, or black for the colon cancer study; the rectal cancer study included Asians and American Indians not living on reservations [20, 21]. Cases had to have tumor registry verification of a first primary adenocarcinoma of the colon or rectum and diagnosed between October 1991 and September 1994 for the colon cancer group and between June 1997 and May 2001 for the rectal cancer group. Tumor tissue was obtained for 97% of all Utah cases diagnosed and for 85% of all KPMCP study participants [22] and included those who signed informed consent and those retrieved by local tumor registries and sent to study investigators without personal identifiers. Local tumor registry data were used to obtain date of birth, date of diagnosis, date of death or date of last contact, and tumor information for those individuals who were not interviewed. The study was approved by the Institutional Review Board of the University of Utah.

### miRNA processing

RNA (miRNA) was extracted from formalin-fixed paraffin embedded tissues. We assessed slides and tumor blocks that were prepared over the duration of the study prior to the time of miRNA isolation to determine their suitability. Older slides produced comparable RNA quality as more recent slides. The study pathologist reviewed slides to delineate tumor, normal, and polyp tissue. Cells were dissected from 1–6 sequential sections on aniline blue stained slides using an H&E slide for reference. Total RNA containing miRNA was extracted, isolated, and purified using the RecoverAll Total Nucleic Acid isolation kit (Ambion), RNA yields were determined using a NanoDrop spectrophotometer. 100 ng total RNA was labeled with cy3 and hybridized to Agilent Human miRNA Microarray V19.0 and were scanned on an Agilent SureScan microarray scanner model G2600D. The Agilent Human microarray was generated using known miRNA sequence information compiled in the Sanger miRBASE database v19.0. The microarray contains probes for 2006 unique human miRNAs, with one to four unique probes for each of the known miRNAs. The miRNA array contains 60,000 unique human sequences and averages 30 replicates per probe sequence. Data were extracted from the scanned image using Agilent Feature Extract software v.11.5.1.1. Data were required to pass stringent QC parameters established by Agilent that included tests for excessive background fluorescence, excessive variation among probe sequence replicates on the array, and measures of the total gene signal on the array to assess low signal. If samples failed to meet quality standards for any of these parameters, the sample was re-labeled, hybridized to arrays, and scanned. If a sample failed QC assessment a second time the sample was deemed to be of poor quality and the individual was excluded from down-stream analysis.

The Agilent platform was found to be highly reliable (*r* = 0.98), to have reasonable agreement with NanoString as well as excellent agreement with qRT-PCR [4, 17]. For unpaired samples due to missing normal scans, we imputed values for normal mucosa as previously described [23]. To minimize differences that could be attributed to the array, amount of RNA, location on array, or other factors that could erroneously influence expression, total gene signal was normalized by multiplying each sample by a scaling factor which was the median of the 75th percentiles of all the samples divided by the 75th percentile of each individual sample [24]. This scaling factor was implemented using SAS 9.4.

### Data sharing and availability

Data will be made available based on limitations of signed informed consent. Because of restrictions of consent forms, data are not incorporated at this time into public data resources. Individuals interested in having access to data can work with study investigators and establish a formal data transfer agreement.

### Statistical methods

Analyses were conducted using the log base 2 transformed data. Data were available for 1855 subjects with survival data. Comparison of stage-specific survival for colon and rectal cancer cases utilized Kaplan Meir test stratified by AJCC tumor stage. This assessment was conducted on all cases diagnosed in Utah and KPMCP during the time period of interest (N for colon cases: AJCC Stage 1 = 503, AJCC Stage 2 = 449; AJCC Stage 3 = 446, and AJCC Stage 4 = 352; N for rectal cases: AJCC Stage 1 = 676, AJCC Stage 2 = 242, AJCC Stage 3 = - 351, and AJCC Stage 4 = 183).

Our analysis included both a discovery component of miRNAs not previously reported with survival as well as a replication component, examining those miRNAs where previous associations were suggested for disease stage or prognosis. In the discovery component of the study we examined over 970 miRNAs and in the replication component of the study we evaluated an additional 105 miRNAs where the miRNA was expressed in at least 5 individuals who had both survival and stage information. In both the discovery and replication components of the study, we analyzed the miRNA data in two groups, one group of more commonly expressed miRNAs (defined as expressed in at least 50% of the population) and a second group less frequently expressed (treated as either expressed or not expressed in the analysis). Since associations from five randomly selected samples of 80% of the population showed that associations with survival for differential expression between carcinoma and normal colorectal mucosa were much more consistent across sample subsets than absolute miRNA carcinoma expression, we used differential expression data when assessing survival.

Survival months were calculated based on difference between the diagnosis date and date of death or date of last follow-up. CRC-specific follow-up included deaths where the primary or secondary cause of death was listed as CRC. Individuals dying of other causes or who were lost to follow-up were censored at their time of death or date of last contact. The R package “survival” was used to calculate *p*-values based upon 10,000 permutations of the likelihood ratio test from the Cox proportional hazards model adjusted for age at diagnosis, gender, AJCC tumor stage, and MSI tumor molecular phenotype. The study population was over 90% non-Hispanic white. Because a number of miRNAs were infrequently expressed, we calculated the HR for these miRNA based upon any vs. no expression using non-permutated *p*-values in SAS 9.4 (SAS Institute, Cary, NC) from the Cox model using the same adjustment variables. We combined the *p*-values of miRNAs in the discovery and replications study components within the two expression level groups to adjust for multiple comparisons using a false discovery rate (FDR) q-value threshold of 0.05 [25].

We have previously assessed these tumors for microsatellite instability (MSI) based on the mononucleotides *BAT26* and *TGFβRII* and a panel of 10 tetranucleotide repeats that were correlated highly with the Bethesda Panel [26]; our study was done prior to the Bethesda Panel development. We report hazard ratios (HR) and 95% Confidence Intervals (CI) from the Cox proportional hazards model after adjusting for age at diagnosis, gender, AJCC tumor stage, and MSI tumor molecular phenotype to assess CRC-specific mortality based on the interquartile range for those more commonly expressed (50% or more of the population) and for any expression vs no expression for less commonly expressed miRNAs. To examine site-specific effects within the colon we created an ordinal site variable and evaluated if there was a significant interaction between miRNAs and site, using a continuous model. Additionally we evaluated if miRNAs were associated significantly with any specific site within the colon.

### Bioinformatics analysis

The top miRNAs that were associated with survival were split into two groups, those that improved survival and those that worsened survival. The target genes for each group were then identified using miRTarBase v6.0 [27]. To better identify which of these target genes might be most relevant for colorectal cancer, we analyzed differential miRNA and mRNA expression between carcinoma and paired normal colorectal mucosa to determine if any miRNAs were affecting mRNA expression in our dataset. For those mRNAs that significantly associated with mRNA, we determined if the mRNA was significantly differentially expressed in colorectal tissue. We then used the significantly differentially expressed mRNAs, with their respective fold changes, as input to QIAGEN's Ingenuity Pathway Analysis (IPA, QIAGEN Redwood City, www.qiagen.com/ingenuity). We used the ‘core analysis' tool, and included direct and indirect relationships, experimentally verified interactions, mammalian species, and all mutations as our analysis settings.

## SUPPLEMENTARY MATERIALS TABLES







## References

[1] Slaby O, Svoboda M, Michalek J, Vyzula R (2009). MicroRNAs in colorectal cancer: translation of molecular biology into clinical application. Mol Cancer.

[2] Amirkhah R, Schmitz U, Linnebacher M, Wolkenhauer O, Farazmand A (2015). MicroRNA-mRNA interactions in colorectal cancer and their role in tumor progression. Genes Chromosomes Cancer.

[3] Iorio MV, Croce CM (2012). Causes and consequences of microRNA dysregulation. Cancer J.

[4] Slattery ML, Herrick JS, Pellatt DF, Stevens JR, Mullany LE, Wolff E, Hoffman MD, Samowitz WS, Wolff RK (2016). MicroRNA profiles in colorectal carcinomas, adenomas and normal colonic mucosa: variations in miRNA expression and disease progression. Carcinogenesis.

[5] Okugawa Y, Toiyama Y, Goel A (2014). An update on microRNAs as colorectal cancer biomarkers: where are we and what's next?. Expert Rev Mol Diagn.

[6] Yang IP, Tsai HL, Huang CW, Huang MY, Hou MF, Juo SH, Wang JY (2013). The functional significance of microRNA-29c in patients with colorectal cancer: a potential circulating biomarker for predicting early relapse. PLoS One.

[7] Tsai HL, Yang IP, Huang CW, Ma CJ, Kuo CH, Lu CY, Juo SH, Wang JY (2013). Clinical significance of micro-RNA-148a in patients with early relapse of stage II stage and III colorectal cancer after curative resection. Transl Res.

[8] Ye TT, Yang YL, Liu XY, Ji QQ, Pan YF, Xiang YQ (2014). Prognostic value of circulating microRNA-21 in digestive system cancers: a meta-analysis. Int J Clin Exp Med.

[9] Schetter AJ, Nguyen GH, Bowman ED, Mathe EA, Yuen ST, Hawkes JE, Croce CM, Leung SY, Harris CC (2009). Association of inflammation-related and microRNA gene expression with cancer-specific mortality of colon adenocarcinoma. Clin Cancer Res.

[10] Huang Z, Huang D, Ni S, Peng Z, Sheng W, Du X (2009). Plasma microRNAs are promising novel biomarkers for early detection of colorectal cancer. Int J Cancer.

[11] Slattery ML, Herrick JS, Mullany LE, Valeri N, Stevens J, Caan BJ, Samowitz W, Wolff RK (2015). An evaluation and replication of miRNAs with disease stage and colorectal cancer-specific mortality. Int J Cancer.

[12] Samowitz WS, Curtin K, Wolff RK, Tripp SR, Caan BJ, Slattery ML (2009). Microsatellite instability and survival in rectal cancer. Cancer Causes Control.

[13] Samowitz WS, Curtin K, Ma KN, Schaffer D, Coleman LW, Leppert M, Slattery ML (2001). Microsatellite instability in sporadic colon cancer is associated with an improved prognosis at the population level. Cancer Epidemiol Biomarkers Prev.

[14] Slattery ML, Herrick JS, Mullany LE, Wolff E, Hoffman MD, Pellatt DF, Stevens JR, Wolff RK (2016). Colorectal tumor molecular phenotype and miRNA: expression profiles and prognosis. Mod Pathol.

[15] Slattery ML, Herrick JS, Pellatt DF, Stevens JR, Mullany LE, Wolff E, Hoffman MD, Samowitz WS, Wolff RK (2016). MicroRNA profiles in colorectal carcinomas, adenomas and normal colonic mucosa: variations in miRNA expression and disease progression. Carcinogenesis.

[16] Lee YC, Lee YL, Chuang JP, Lee JC (2013). Differences in survival between colon and rectal cancer from SEER data. PLoS One.

[17] Pellatt DF, Stevens JR, Wolff RK, Mullany LE, Herrick JS, Samowitz W, Slattery ML (2016). Expression Profiles of miRNA Subsets Distinguish Human Colorectal Carcinoma and Normal Colonic Mucosa. Clin Transl Gastroenterol.

[18] Landskroner-Eiger SMI, Sessa WC (2013). miRNAS as modulators of angiogenesis. Cold Spring Harb Perspect Med.

[19] Watnick RS (2012). The role of tumor microenvironment in regulating angiogenesis. Cold Spring Harb Perspect Med.

[20] Slattery ML, Potter J, Caan B, Edwards S, Coates A, Ma KN, Berry TD (1997). Energy balance and colon cancer—beyond physical activity. Cancer Res.

[21] Slattery ML, Caan BJ, Benson J, Murtaugh M (2003). Energy balance and rectal cancer: an evaluation of energy intake, energy expenditure, and body mass index. Nutr Cancer.

[22] Slattery ML, Edwards SL, Palmer L, Curtin K, Morse J, Anderson K, Samowitz W (2000). Use of archival tissue in epidemiologic studies: collection procedures and assessment of potential sources of bias. Mutat Res.

[23] Suyundikov A, Stevens JR, Corcoran C, Herrick J, Wolff RK, Slattery ML (2015). Accounting for Dependence Induced by Weighted KNN Imputation in Paired Samples, Motivated by a Colorectal Cancer Study. PloS one.

[24] Agilent Technologies I (2013). Agilent GeneSpring User Manual.

[25] Storey JD, Tibshirani R (2003). Statistical significance for genomewide studies. Proc Natl Acad Sci USA.

[26] Slattery ML, Curtin K, Anderson K, Ma KN, Ballard L, Edwards S, Schaffer D, Potter J, Leppert M, Samowitz WS (2000). Associations between cigarette smoking, lifestyle factors, and microsatellite instability in colon tumors. J Natl Cancer Inst.

[27] Chou CH, Chang NW, Shrestha S, Hsu SD, Lin YL, Lee WH, Yang CD, Hong HC, Wei TY, Tu SJ, Tsai TR, Ho SY (2016). miRTarBase 2016: updates to the experimentally validated miRNA-target interactions database. Nucleic Acids Res.

