# Supplementary material for: Site-specific associations between miRNA expression and survival in colorectal cancer cases
Source: Oncotarget. 2016 Aug 10;7(37):60193–205. doi: 10.18632/oncotarget.11173 (PMC5312378; doi:10.18632/oncotarget.11173)
Supplement: Supplementary file 1 [file oncotarget-07-60193-s001.pdf]

Site-specific associations between miRNA expression and survival in colorectal cancer cases

Supplementary Materials

Supplementary Table S1: Colon differential expression by AJCC stage (adjusted for age, sex, and MSI)

Stage	miRNA	% expressing	Q1	Q3	HR	95% (CI)	*p*-value	*q*-value
1&2	hsa-miR-181d	98.0	–0.06	0.54	0.89	(0.74, 1.08)	0.02	1.00
	hsa-miR-30b-3p	90.7	–0.09	0.34	0.91	(0.82, 1.00)	0.02	1.00
	hsa-miR-4284	100.0	–0.41	0.51	0.69	(0.54, 0.90)	0.01	1.00
	hsa-miR-429	65.9	–0.22	1.69	0.80	(0.64, 0.99)	0.04	1.00
	hsa-miR-4461	65.7	–0.48	0.69	0.80	(0.66, 0.97)	0.01	1.00
	hsa-miR-4485	100.0	–0.80	0.08	0.66	(0.47, 0.93)	0.04	1.00
	hsa-miR-758-5p	99.7	–0.48	0.15	0.75	(0.60, 0.93)	0.04	1.00
3&4	hsa-miR-15a-5p	62.8	–0.88	1.45	0.83	(0.71, 0.97)	0.02	1.00
	hsa-miR-181d	98.0	–0.07	0.57	1.11	(1.02, 1.20)	0.03	1.00
	hsa-miR-192-5p	97.3	–1.59	-0.09	0.88	(0.79, 0.97)	0.01	0.97
	hsa-miR-194-3p	100.0	–0.64	0.02	0.86	(0.74, 0.99)	0.05	1.00
	hsa-miR-1973	99.9	–0.82	0.18	0.90	(0.83, 0.99)	0.03	1.00
	hsa-miR-30a-5p	31.0	–1.60	0.00	1.15	(1.02, 1.30)	0.03	1.00
	hsa-miR-30b-3p	90.7	–0.10	0.29	1.06	(1.01, 1.11)	0.01	1.00
	hsa-miR-3161	99.4	–0.29	0.06	1.14	(1.03, 1.25)	0.01	0.97
	hsa-miR-3622b-5p	100.0	–0.21	0.14	0.87	(0.78, 0.98)	0.02	1.00
	hsa-miR-3651	99.3	0.53	1.59	0.91	(0.82, 1.00)	0.04	1.00
	hsa-miR-3676-3p	72.6	–0.47	0.52	0.92	(0.85, 0.99)	0.03	1.00
	hsa-miR-4269	97.0	–0.14	0.24	1.05	(1.00, 1.10)	0.04	1.00
	hsa-miR-4284	100.0	–0.63	0.37	0.84	(0.75, 0.95)	0.00	0.97
	hsa-miR-4485	100.0	–0.89	0.03	0.83	(0.71, 0.96)	0.01	1.00
	hsa-miR-492	95.4	–0.78	0.12	1.16	(1.05, 1.28)	0.00	0.97
	hsa-miR-662	99.9	–0.34	0.01	0.91	(0.83, 0.99)	0.05	1.00
	hsa-miR-99b-5p	47.3	–0.45	1.54	1.23	(1.08, 1.40)	0.00	0.97

Supplementary Table S2: Rectal differential expression by AJCC stage (adjusted for age, sex, and MSI). See Supplementary_Table_S2

Supplementary Table S3: Hazard ratio (HR) associated with miRNA expression for colon cancer adjusted for age, sex, and MSI status

Study	% expressing	25th%ile	75th%ile	HR	95% (CI)	*p*-value	*q*-value
hsa-miR-15a-5p	62.9	–0.51	1.45	0.87	(0.77, 0.97)	0.0153	0.96
hsa-miR-181a-5p	99.8	–0.12	0.75	1.15	(1.01, 1.31)	0.0396	1.00
hsa-miR-192-5p	99.4	–1.46	–0.02	0.87	(0.80, 0.95)	0.0031	0.51
hsa-miR-193b-3p	83.2	–0.19	1.65	1.13	(1.00, 1.27)	0.0395	1.00
hsa-miR-194-3p	99.9	–0.60	0.03	0.85	(0.75, 0.97)	0.0322	1.00
hsa-miR-194-5p	99.6	–1.28	0.09	0.90	(0.82, 0.99)	0.0404	1.00
hsa-miR-196b-5p	71.0	–0.57	2.27	0.85	(0.74, 0.98)	0.0417	1.00
hsa-miR-1973	100.0	–0.74	0.21	0.90	(0.83, 0.97)	0.0104	0.91
hsa-miR-30a-5p	59.4	–1.64	0.00	1.13	(1.01, 1.27)	0.0274	1.00
hsa-miR-3161	99.8	–0.28	0.06	1.11	(1.02, 1.21)	0.0107	0.91
hsa-miR-3622b-5p	100.0	–0.22	0.15	0.87	(0.78, 0.97)	0.0166	0.96
hsa-miR-3651	99.1	0.51	1.62	0.90	(0.82, 0.98)	0.0230	1.00
hsa-miR-3676-3p	76.4	–0.46	0.47	0.93	(0.87, 0.99)	0.0229	1.00
hsa-miR-4284	100.0	–0.52	0.49	0.82	(0.74, 0.91)	0.0004	0.28
hsa-miR-4485	100.0	–0.83	0.06	0.81	(0.71, 0.92)	0.0019	0.46
hsa-miR-492	98.4	–0.81	0.13	1.14	(1.04, 1.25)	0.0037	0.51
hsa-miR-497-5p	71.6	–2.35	0.00	1.18	(1.00, 1.39)	0.0494	1.00
hsa-miR-662	99.9	–0.36	0.01	0.91	(0.83, 0.99)	0.0350	1.00
hsa-miR-99b-5p	53.2	–0.46	1.15	1.16	(1.06, 1.28)	0.0020	0.46

Supplementary Table S4: Differential miRNA expression between carcinoma and normal mucosa associated with survival in rectal cancer cases where FDR *q* value is ≥ 0.031. See Supplementary_Table_S4
