# Supplementary material for: Site-specific associations between miRNA expression and survival in colorectal cancer cases
Source: Oncotarget. 2016 Aug 10;7(37):60193–205. doi: 10.18632/oncotarget.11173 (PMC5312378; doi:10.18632/oncotarget.11173)
Supplement: Supplementary file 2 [file oncotarget-07-60193-s002.docx]

| Supplemental Table 2. Rectal differential expression by AJCC stage (adjusted for age, sex, and MSI). | | | | | | | | |  |
| --- | --- | --- | --- | --- | --- | --- | --- | --- | --- |
| Stage | miRNA | % expressing | Q1 | Q3 | HR | 95% (CI) | | p-value | q-value |
| 1&2 | hsa-miR-1228-5p | 96.6 | -0.17 | 0.41 | 0.82 | (0.71, | 0.96) | 0.02 | 0.294 |
|  | hsa-miR-1291 | 76.3 | 0.00 | 1.40 | 0.76 | (0.61, | 0.94) | 0.02 | 0.294 |
|  | hsa-miR-129-5p | 98.6 | -0.44 | 0.00 | 1.28 | (1.05, | 1.57) | 0.02 | 0.294 |
|  | hsa-miR-130a-3p | 52.4 | -0.61 | 1.60 | 0.74 | (0.57, | 0.97) | 0.04 | 0.294 |
|  | hsa-miR-139-3p | 99.6 | -0.57 | -0.20 | 1.31 | (1.05, | 1.64) | 0.02 | 0.294 |
|  | hsa-miR-15a-5p | 62.8 | -0.04 | 1.69 | 0.68 | (0.53, | 0.86) | 0.00 | 0.294 |
|  | hsa-miR-26a-5p | 99.2 | -0.34 | 0.63 | 0.76 | (0.58, | 0.99) | 0.04 | 0.294 |
|  | hsa-miR-30e-5p | 46.8 | -1.51 | 0.39 | 0.71 | (0.52, | 0.97) | 0.04 | 0.294 |
|  | hsa-miR-3185 | 99.9 | -0.30 | 0.16 | 1.29 | (1.02, | 1.63) | 0.05 | 0.294 |
|  | hsa-miR-3187-3p | 97.7 | -0.32 | 0.15 | 1.21 | (1.04, | 1.40) | 0.02 | 0.294 |
|  | hsa-miR-3188 | 100.0 | -0.56 | -0.06 | 1.31 | (1.02, | 1.68) | 0.05 | 0.294 |
|  | hsa-miR-361-5p | 88.6 | 0.18 | 1.59 | 0.75 | (0.60, | 0.94) | 0.02 | 0.294 |
|  | hsa-miR-3666 | 98.9 | -0.35 | 0.04 | 1.29 | (1.04, | 1.60) | 0.02 | 0.294 |
|  | hsa-miR-3679-5p | 100.0 | -0.49 | 0.02 | 1.29 | (1.01, | 1.65) | 0.05 | 0.294 |
|  | hsa-miR-422a | 98.4 | -0.26 | 0.11 | 1.19 | (1.02, | 1.38) | 0.02 | 0.294 |
|  | hsa-miR-429 | 65.9 | 0.00 | 2.06 | 0.77 | (0.62, | 0.96) | 0.03 | 0.294 |
|  | hsa-miR-4299 | 100.0 | -0.51 | -0.01 | 1.29 | (1.05, | 1.60) | 0.02 | 0.294 |
|  | hsa-miR-4323 | 97.9 | -1.01 | -0.36 | 1.29 | (1.03, | 1.61) | 0.02 | 0.294 |
|  | hsa-miR-4450 | 65.6 | -0.16 | 0.85 | 0.84 | (0.71, | 0.98) | 0.03 | 0.294 |
|  | hsa-miR-4461 | 65.7 | -0.35 | 0.79 | 0.81 | (0.68, | 0.98) | 0.03 | 0.294 |
|  | hsa-miR-4470 | 100.0 | -0.41 | -0.08 | 1.32 | (1.05, | 1.65) | 0.02 | 0.294 |
|  | hsa-miR-4505 | 100.0 | -0.46 | 0.03 | 1.32 | (1.05, | 1.65) | 0.02 | 0.294 |
|  | hsa-miR-4507 | 100.0 | -0.50 | 0.01 | 1.28 | (1.02, | 1.60) | 0.05 | 0.294 |
|  | hsa-miR-4660 | 70.3 | -0.08 | 0.76 | 0.87 | (0.76, | 0.99) | 0.04 | 0.294 |
|  | hsa-miR-4687-3p | 100.0 | -0.60 | -0.05 | 1.33 | (1.04, | 1.70) | 0.04 | 0.294 |
|  | hsa-miR-4725-3p | 97.4 | -0.41 | 0.04 | 1.25 | (1.01, | 1.54) | 0.03 | 0.294 |
|  | hsa-miR-4749-3p | 95.9 | -0.95 | -0.34 | 1.36 | (1.08, | 1.70) | 0.01 | 0.294 |
|  | hsa-miR-4787-5p | 100.0 | -0.74 | -0.13 | 1.34 | (1.05, | 1.72) | 0.03 | 0.294 |
|  | hsa-miR-489 | 96.9 | -0.30 | 0.11 | 1.17 | (1.04, | 1.31) | 0.02 | 0.294 |
|  | hsa-miR-5001-5p | 100.0 | -0.66 | -0.07 | 1.30 | (1.02, | 1.65) | 0.05 | 0.294 |
|  | hsa-miR-5010-5p | 97.4 | -0.17 | 0.18 | 0.89 | (0.81, | 0.97) | 0.01 | 0.294 |
|  | hsa-miR-501-3p | 96.0 | 0.57 | 1.76 | 0.72 | (0.55, | 0.93) | 0.02 | 0.294 |
|  | hsa-miR-508-5p | 80.4 | -0.43 | 0.12 | 0.83 | (0.74, | 0.94) | 0.01 | 0.294 |
|  | hsa-miR-5093 | 70.8 | -0.51 | 0.36 | 0.83 | (0.71, | 0.97) | 0.02 | 0.294 |
|  | hsa-miR-5195-5p | 85.9 | -0.13 | 0.60 | 0.86 | (0.77, | 0.95) | 0.01 | 0.294 |
|  | hsa-miR-548q | 100.0 | -0.79 | -0.17 | 1.38 | (1.07, | 1.78) | 0.02 | 0.294 |
|  | hsa-miR-5572 | 82.7 | -0.28 | 0.16 | 0.93 | (0.86, | 1.00) | 0.04 | 0.294 |
|  | hsa-miR-6074 | 99.3 | -0.16 | 0.14 | 1.18 | (1.04, | 1.33) | 0.02 | 0.294 |
|  | hsa-miR-6124 | 100.0 | -0.34 | 0.19 | 1.32 | (1.05, | 1.67) | 0.03 | 0.294 |
|  | hsa-miR-6125 | 100.0 | -0.65 | -0.10 | 1.30 | (1.02, | 1.66) | 0.05 | 0.294 |
|  | hsa-miR-636 | 99.4 | -0.59 | 0.09 | 1.29 | (1.02, | 1.63) | 0.03 | 0.294 |
|  | hsa-miR-650 | 77.7 | -2.67 | -1.22 | 0.79 | (0.60, | 1.03) | 0.05 | 0.294 |
|  | hsa-miR-654-5p | 99.8 | -0.68 | -0.18 | 1.34 | (1.06, | 1.70) | 0.02 | 0.294 |
|  | hsa-miR-6722-3p | 100.0 | -0.44 | 0.05 | 1.31 | (1.02, | 1.68) | 0.05 | 0.294 |
| 3&4 | hsa-miR-1182 | 99.8 | -0.10 | 0.21 | 1.26 | (1.11, | 1.44) | 0.00 | 0.073 |
|  | hsa-miR-1183 | 100.0 | -0.16 | 0.11 | 1.20 | (1.04, | 1.39) | 0.02 | 0.091 |
|  | hsa-miR-1203 | 43.1 | -1.59 | 0.00 | 0.80 | (0.64, | 1.00) | 0.03 | 0.096 |
|  | hsa-miR-1207-5p | 100.0 | -0.45 | 0.01 | 1.22 | (1.03, | 1.45) | 0.03 | 0.097 |
|  | hsa-miR-1225-5p | 100.0 | -0.42 | -0.01 | 1.22 | (1.03, | 1.44) | 0.03 | 0.096 |
|  | hsa-miR-1226-5p | 100.0 | -0.31 | 0.08 | 1.21 | (1.02, | 1.42) | 0.03 | 0.096 |
|  | hsa-miR-1228-5p | 96.6 | -0.17 | 0.40 | 0.85 | (0.75, | 0.96) | 0.01 | 0.076 |
|  | hsa-miR-1229-5p | 100.0 | -0.40 | 0.08 | 1.26 | (1.06, | 1.49) | 0.01 | 0.078 |
|  | hsa-miR-1233-1-5p | 100.0 | -0.37 | -0.02 | 1.32 | (1.11, | 1.58) | 0.00 | 0.074 |
|  | hsa-miR-1234-5p | 100.0 | -0.48 | -0.05 | 1.20 | (1.02, | 1.42) | 0.04 | 0.098 |
|  | hsa-miR-1288 | 99.9 | -0.52 | 0.02 | 1.36 | (1.11, | 1.65) | 0.00 | 0.073 |
|  | hsa-miR-129-5p | 98.6 | -0.42 | 0.02 | 1.20 | (1.05, | 1.37) | 0.02 | 0.084 |
|  | hsa-miR-1305 | 100.0 | -0.57 | -0.03 | 1.31 | (1.08, | 1.59) | 0.01 | 0.076 |
|  | hsa-miR-130a-3p | 52.4 | -1.08 | 1.59 | 0.73 | (0.57, | 0.95) | 0.01 | 0.078 |
|  | hsa-miR-134 | 100.0 | -0.40 | -0.01 | 1.20 | (1.02, | 1.41) | 0.04 | 0.098 |
|  | hsa-miR-142-3p | 30.8 | -1.69 | 0.00 | 0.79 | (0.65, | 0.97) | 0.02 | 0.091 |
|  | hsa-miR-146a-5p | 70.2 | -0.85 | 1.23 | 0.75 | (0.62, | 0.90) | 0.00 | 0.073 |
|  | hsa-miR-1470 | 76.5 | -0.88 | 0.01 | 0.87 | (0.77, | 0.99) | 0.03 | 0.096 |
|  | hsa-miR-150-3p | 100.0 | -0.31 | 0.02 | 1.20 | (1.02, | 1.40) | 0.03 | 0.096 |
|  | hsa-miR-187-5p | 97.7 | -0.52 | 0.01 | 1.14 | (1.01, | 1.28) | 0.05 | 0.109 |
|  | hsa-miR-188-5p | 100.0 | -0.45 | -0.02 | 1.19 | (1.01, | 1.39) | 0.05 | 0.109 |
|  | hsa-miR-1914-3p | 100.0 | -0.34 | -0.01 | 1.22 | (1.03, | 1.44) | 0.03 | 0.096 |
|  | hsa-miR-1915-3p | 100.0 | -0.60 | -0.05 | 1.29 | (1.07, | 1.56) | 0.01 | 0.079 |
|  | hsa-miR-196b-5p | 76.8 | 0.00 | 3.14 | 0.73 | (0.56, | 0.94) | 0.02 | 0.091 |
|  | hsa-miR-198 | 100.0 | -0.30 | 0.09 | 1.28 | (1.04, | 1.57) | 0.03 | 0.096 |
|  | hsa-miR-19b-3p | 93.1 | 0.78 | 2.35 | 0.79 | (0.68, | 0.93) | 0.01 | 0.074 |
|  | hsa-miR-202-3p | 99.8 | -0.27 | 0.07 | 1.30 | (1.12, | 1.49) | 0.00 | 0.073 |
|  | hsa-miR-2392 | 100.0 | -0.27 | 0.16 | 1.18 | (1.01, | 1.36) | 0.04 | 0.101 |
|  | hsa-miR-2467-3p | 99.9 | -0.03 | 0.28 | 1.23 | (1.04, | 1.45) | 0.02 | 0.092 |
|  | hsa-miR-30c-1-3p | 99.9 | -0.34 | -0.01 | 1.33 | (1.12, | 1.58) | 0.00 | 0.073 |
|  | hsa-miR-30e-5p | 46.8 | -1.57 | 0.51 | 0.66 | (0.51, | 0.87) | 0.00 | 0.073 |
|  | hsa-miR-3125 | 100.0 | -0.41 | 0.05 | 1.31 | (1.08, | 1.58) | 0.01 | 0.076 |
|  | hsa-miR-3127-5p | 100.0 | -0.43 | -0.03 | 1.26 | (1.07, | 1.49) | 0.01 | 0.076 |
|  | hsa-miR-3138 | 100.0 | -0.27 | 0.04 | 1.19 | (1.02, | 1.39) | 0.03 | 0.097 |
|  | hsa-miR-3188 | 100.0 | -0.50 | -0.07 | 1.22 | (1.03, | 1.44) | 0.03 | 0.097 |
|  | hsa-miR-3195 | 100.0 | -0.59 | -0.03 | 1.31 | (1.08, | 1.59) | 0.00 | 0.074 |
|  | hsa-miR-3196 | 100.0 | -0.49 | -0.06 | 1.25 | (1.02, | 1.53) | 0.03 | 0.096 |
|  | hsa-miR-3198 | 100.0 | -0.33 | 0.13 | 1.36 | (1.13, | 1.64) | 0.00 | 0.073 |
|  | hsa-miR-3200-5p | 99.7 | -0.41 | -0.04 | 1.28 | (1.07, | 1.55) | 0.01 | 0.076 |
|  | hsa-miR-3202 | 99.7 | -0.24 | 0.12 | 1.18 | (1.01, | 1.39) | 0.05 | 0.109 |
|  | hsa-miR-324-3p | 100.0 | 0.08 | 0.52 | 1.21 | (1.00, | 1.47) | 0.04 | 0.104 |
|  | hsa-miR-345-3p | 100.0 | -0.21 | 0.22 | 1.19 | (1.00, | 1.41) | 0.04 | 0.104 |
|  | hsa-miR-3609 | 45.3 | -0.44 | 1.07 | 0.78 | (0.64, | 0.94) | 0.01 | 0.076 |
|  | hsa-miR-3610 | 100.0 | -0.54 | -0.03 | 1.24 | (1.04, | 1.48) | 0.02 | 0.096 |
|  | hsa-miR-3621 | 100.0 | -0.34 | 0.04 | 1.24 | (1.06, | 1.46) | 0.01 | 0.078 |
|  | hsa-miR-3622b-5p | 100.0 | -0.17 | 0.17 | 1.23 | (1.02, | 1.49) | 0.03 | 0.096 |
|  | hsa-miR-3652 | 100.0 | -0.29 | 0.05 | 1.22 | (1.02, | 1.46) | 0.04 | 0.098 |
|  | hsa-miR-3663-3p | 100.0 | -0.40 | 0.05 | 1.22 | (1.02, | 1.46) | 0.04 | 0.104 |
|  | hsa-miR-3665 | 100.0 | -0.53 | -0.01 | 1.22 | (1.01, | 1.48) | 0.05 | 0.109 |
|  | hsa-miR-3667-5p | 99.6 | -0.30 | 0.12 | 1.26 | (1.10, | 1.45) | 0.00 | 0.073 |
|  | hsa-miR-3679-5p | 100.0 | -0.46 | 0.05 | 1.25 | (1.04, | 1.50) | 0.02 | 0.093 |
|  | hsa-miR-378b | 99.9 | -0.13 | 0.16 | 1.24 | (1.05, | 1.47) | 0.01 | 0.076 |
|  | hsa-miR-3917 | 100.0 | -0.24 | 0.11 | 1.19 | (1.00, | 1.43) | 0.05 | 0.109 |
|  | hsa-miR-3937 | 100.0 | -0.32 | 0.06 | 1.21 | (1.04, | 1.42) | 0.02 | 0.091 |
|  | hsa-miR-3945 | 99.9 | -0.38 | -0.03 | 1.24 | (1.05, | 1.47) | 0.01 | 0.078 |
|  | hsa-miR-3960 | 100.0 | -0.48 | 0.12 | 1.29 | (1.06, | 1.57) | 0.02 | 0.084 |
|  | hsa-miR-4253 | 100.0 | -0.27 | 0.10 | 1.32 | (1.09, | 1.60) | 0.01 | 0.074 |
|  | hsa-miR-425-3p | 97.5 | -0.59 | 0.12 | 1.21 | (1.03, | 1.42) | 0.03 | 0.096 |
|  | hsa-miR-4270 | 100.0 | -0.28 | 0.12 | 1.19 | (1.01, | 1.39) | 0.05 | 0.109 |
|  | hsa-miR-4281 | 100.0 | -0.39 | 0.13 | 1.24 | (1.03, | 1.49) | 0.03 | 0.096 |
|  | hsa-miR-4294 | 99.8 | -0.18 | 0.11 | 1.29 | (1.09, | 1.53) | 0.00 | 0.073 |
|  | hsa-miR-4298 | 100.0 | -0.04 | 0.33 | 1.18 | (1.00, | 1.40) | 0.05 | 0.109 |
|  | hsa-miR-431-5p | 99.6 | -0.61 | -0.11 | 1.20 | (1.07, | 1.35) | 0.01 | 0.074 |
|  | hsa-miR-4429 | 100.0 | -0.25 | 0.14 | 1.19 | (1.03, | 1.37) | 0.03 | 0.097 |
|  | hsa-miR-4443 | 100.0 | -0.59 | 0.06 | 1.28 | (1.07, | 1.54) | 0.01 | 0.076 |
|  | hsa-miR-4459 | 100.0 | -0.56 | -0.09 | 1.27 | (1.08, | 1.49) | 0.01 | 0.074 |
|  | hsa-miR-4461 | 65.7 | -1.07 | 0.37 | 0.81 | (0.69, | 0.95) | 0.01 | 0.076 |
|  | hsa-miR-4463 | 100.0 | -0.29 | 0.11 | 1.20 | (1.01, | 1.42) | 0.04 | 0.104 |
|  | hsa-miR-4466 | 100.0 | -0.48 | 0.01 | 1.23 | (1.03, | 1.47) | 0.03 | 0.096 |
|  | hsa-miR-4470 | 100.0 | -0.42 | -0.09 | 1.25 | (1.05, | 1.48) | 0.01 | 0.082 |
|  | hsa-miR-4472 | 94.7 | -0.43 | 0.12 | 1.14 | (1.02, | 1.27) | 0.02 | 0.096 |
|  | hsa-miR-4476 | 100.0 | -0.28 | 0.01 | 1.17 | (1.02, | 1.34) | 0.03 | 0.096 |
|  | hsa-miR-4481 | 100.0 | -0.27 | 0.05 | 1.17 | (1.03, | 1.34) | 0.02 | 0.092 |
|  | hsa-miR-4499 | 100.0 | -0.46 | -0.02 | 1.29 | (1.08, | 1.54) | 0.01 | 0.076 |
|  | hsa-miR-4505 | 100.0 | -0.43 | 0.09 | 1.23 | (1.01, | 1.48) | 0.04 | 0.097 |
|  | hsa-miR-4508 | 100.0 | -0.50 | -0.11 | 1.20 | (1.02, | 1.42) | 0.04 | 0.097 |
|  | hsa-miR-4514 | 99.9 | -0.24 | 0.10 | 1.24 | (1.04, | 1.46) | 0.02 | 0.084 |
|  | hsa-miR-4516 | 100.0 | -0.53 | -0.05 | 1.24 | (1.03, | 1.49) | 0.03 | 0.096 |
|  | hsa-miR-4526 | 72.7 | -0.28 | 0.11 | 0.95 | (0.89, | 1.00) | 0.04 | 0.104 |
|  | hsa-miR-4534 | 100.0 | -0.49 | -0.01 | 1.24 | (1.05, | 1.48) | 0.02 | 0.091 |
|  | hsa-miR-4539 | 100.0 | -0.81 | -0.41 | 1.19 | (1.01, | 1.39) | 0.05 | 0.105 |
|  | hsa-miR-4634 | 100.0 | -0.56 | 0.08 | 1.26 | (1.02, | 1.54) | 0.04 | 0.101 |
|  | hsa-miR-4654 | 49.8 | -0.04 | 1.00 | 0.87 | (0.76, | 0.98) | 0.03 | 0.096 |
|  | hsa-miR-4655-5p | 100.0 | -0.22 | 0.18 | 1.27 | (1.07, | 1.52) | 0.01 | 0.074 |
|  | hsa-miR-4657 | 61.3 | 0.00 | 1.05 | 0.80 | (0.69, | 0.94) | 0.00 | 0.074 |
|  | hsa-miR-4659a-3p | 99.4 | -0.30 | 0.13 | 1.27 | (1.08, | 1.50) | 0.01 | 0.076 |
|  | hsa-miR-4673 | 100.0 | -0.47 | -0.07 | 1.23 | (1.04, | 1.46) | 0.03 | 0.096 |
|  | hsa-miR-4687-3p | 100.0 | -0.50 | -0.04 | 1.24 | (1.05, | 1.47) | 0.01 | 0.082 |
|  | hsa-miR-4690-5p | 100.0 | -0.66 | -0.15 | 1.22 | (1.02, | 1.46) | 0.03 | 0.096 |
|  | hsa-miR-4697-5p | 99.9 | -0.46 | 0.00 | 1.21 | (1.02, | 1.44) | 0.04 | 0.098 |
|  | hsa-miR-4707-3p | 80.0 | -0.89 | -0.08 | 1.20 | (1.06, | 1.37) | 0.01 | 0.078 |
|  | hsa-miR-4713-3p | 100.0 | -0.52 | 0.03 | 1.36 | (1.11, | 1.68) | 0.01 | 0.074 |
|  | hsa-miR-4716-3p | 100.0 | -0.50 | -0.01 | 1.36 | (1.12, | 1.65) | 0.00 | 0.073 |
|  | hsa-miR-4717-3p | 80.3 | -0.16 | 0.23 | 0.94 | (0.89, | 1.00) | 0.04 | 0.104 |
|  | hsa-miR-4730 | 47.0 | -1.20 | 0.37 | 0.84 | (0.73, | 0.97) | 0.02 | 0.087 |
|  | hsa-miR-4738-3p | 100.0 | -0.18 | 0.11 | 1.18 | (1.01, | 1.38) | 0.04 | 0.104 |
|  | hsa-miR-4739 | 100.0 | -0.48 | -0.02 | 1.24 | (1.03, | 1.49) | 0.03 | 0.096 |
|  | hsa-miR-4749-5p | 100.0 | -0.15 | 0.12 | 1.19 | (1.01, | 1.41) | 0.04 | 0.103 |
|  | hsa-miR-4783-3p | 99.8 | -0.41 | 0.00 | 1.28 | (1.05, | 1.56) | 0.02 | 0.091 |
|  | hsa-miR-4784 | 99.8 | -0.25 | 0.01 | 1.19 | (1.05, | 1.35) | 0.01 | 0.078 |
|  | hsa-miR-4793-3p | 75.1 | -0.47 | 0.39 | 0.90 | (0.82, | 0.99) | 0.03 | 0.096 |
|  | hsa-miR-4793-5p | 100.0 | -0.12 | 0.19 | 1.21 | (1.02, | 1.44) | 0.03 | 0.096 |
|  | hsa-miR-486-5p | 99.7 | -0.62 | -0.17 | 1.23 | (1.02, | 1.49) | 0.03 | 0.096 |
|  | hsa-miR-493-3p | 69.6 | -0.69 | 0.56 | 0.80 | (0.66, | 0.96) | 0.02 | 0.091 |
|  | hsa-miR-5006-5p | 100.0 | -0.33 | -0.04 | 1.21 | (1.03, | 1.43) | 0.02 | 0.096 |
|  | hsa-miR-5088 | 100.0 | -0.40 | 0.01 | 1.28 | (1.07, | 1.54) | 0.01 | 0.076 |
|  | hsa-miR-5100 | 100.0 | -0.70 | 0.02 | 1.36 | (1.10, | 1.69) | 0.00 | 0.074 |
|  | hsa-miR-5194 | 100.0 | -0.41 | 0.00 | 1.24 | (1.05, | 1.46) | 0.01 | 0.082 |
|  | hsa-miR-519e-5p | 44.1 | -0.22 | 0.14 | 0.95 | (0.90, | 1.00) | 0.03 | 0.097 |
|  | hsa-miR-550a-3-5p | 100.0 | -0.19 | 0.14 | 1.31 | (1.07, | 1.59) | 0.01 | 0.076 |
|  | hsa-miR-550b-2-5p | 99.9 | -0.33 | 0.02 | 1.26 | (1.08, | 1.48) | 0.01 | 0.076 |
|  | hsa-miR-5581-5p | 100.0 | -0.51 | -0.03 | 1.33 | (1.09, | 1.61) | 0.01 | 0.074 |
|  | hsa-miR-5703 | 100.0 | -0.64 | 0.00 | 1.25 | (1.07, | 1.48) | 0.01 | 0.076 |
|  | hsa-miR-572 | 100.0 | -0.66 | -0.08 | 1.28 | (1.06, | 1.54) | 0.01 | 0.084 |
|  | hsa-miR-575 | 100.0 | -0.36 | 0.08 | 1.23 | (1.02, | 1.49) | 0.05 | 0.109 |
|  | hsa-miR-584-5p | 100.0 | -0.29 | 0.02 | 1.16 | (1.01, | 1.34) | 0.03 | 0.097 |
|  | hsa-miR-601 | 100.0 | -0.33 | 0.00 | 1.19 | (1.04, | 1.37) | 0.03 | 0.096 |
|  | hsa-miR-6068 | 100.0 | -0.65 | -0.08 | 1.25 | (1.04, | 1.51) | 0.03 | 0.096 |
|  | hsa-miR-6069 | 100.0 | -0.33 | 0.16 | 1.19 | (1.01, | 1.39) | 0.03 | 0.096 |
|  | hsa-miR-6076 | 100.0 | -0.33 | 0.03 | 1.21 | (1.02, | 1.43) | 0.04 | 0.097 |
|  | hsa-miR-6085 | 100.0 | -0.28 | 0.08 | 1.15 | (0.99, | 1.34) | 0.02 | 0.096 |
|  | hsa-miR-6086 | 100.0 | -0.37 | 0.06 | 1.21 | (1.01, | 1.45) | 0.04 | 0.104 |
|  | hsa-miR-6087 | 100.0 | -0.49 | -0.04 | 1.24 | (1.04, | 1.49) | 0.02 | 0.096 |
|  | hsa-miR-6088 | 100.0 | -0.41 | 0.09 | 1.27 | (1.06, | 1.52) | 0.01 | 0.078 |
|  | hsa-miR-6089 | 100.0 | -0.55 | 0.00 | 1.25 | (1.03, | 1.52) | 0.03 | 0.096 |
|  | hsa-miR-6090 | 100.0 | -0.52 | 0.06 | 1.27 | (1.05, | 1.54) | 0.02 | 0.087 |
|  | hsa-miR-6124 | 100.0 | -0.27 | 0.28 | 1.26 | (1.04, | 1.51) | 0.02 | 0.093 |
|  | hsa-miR-6125 | 100.0 | -0.61 | -0.07 | 1.24 | (1.03, | 1.51) | 0.04 | 0.097 |
|  | hsa-miR-6126 | 100.0 | -0.55 | -0.11 | 1.25 | (1.05, | 1.49) | 0.02 | 0.087 |
|  | hsa-miR-6131 | 100.0 | -0.58 | -0.04 | 1.38 | (1.13, | 1.69) | 0.00 | 0.073 |
|  | hsa-miR-6132 | 100.0 | -0.35 | 0.12 | 1.23 | (1.02, | 1.49) | 0.03 | 0.096 |
|  | hsa-miR-6165 | 100.0 | -0.05 | 0.36 | 1.21 | (1.02, | 1.44) | 0.04 | 0.097 |
|  | hsa-miR-622 | 100.0 | -0.22 | 0.14 | 1.19 | (1.02, | 1.39) | 0.03 | 0.096 |
|  | hsa-miR-630 | 100.0 | -0.66 | -0.04 | 1.28 | (1.08, | 1.52) | 0.01 | 0.074 |
|  | hsa-miR-638 | 100.0 | -0.57 | -0.06 | 1.23 | (1.02, | 1.48) | 0.04 | 0.101 |
|  | hsa-miR-6512-5p | 99.9 | -0.37 | 0.05 | 1.22 | (1.03, | 1.44) | 0.03 | 0.096 |
|  | hsa-miR-662 | 99.9 | -0.33 | 0.03 | 1.19 | (1.08, | 1.32) | 0.01 | 0.074 |
|  | hsa-miR-6717-5p | 100.0 | -0.54 | 0.01 | 1.39 | (1.15, | 1.69) | 0.00 | 0.073 |
|  | hsa-miR-671-5p | 100.0 | -0.42 | 0.06 | 1.21 | (1.03, | 1.44) | 0.01 | 0.076 |
|  | hsa-miR-6722-3p | 100.0 | -0.35 | 0.09 | 1.30 | (1.09, | 1.55) | 0.01 | 0.074 |
|  | hsa-miR-6724-5p | 100.0 | -0.34 | 0.11 | 1.22 | (1.02, | 1.46) | 0.04 | 0.104 |
|  | hsa-miR-760 | 100.0 | -0.23 | 0.11 | 1.17 | (1.02, | 1.34) | 0.03 | 0.096 |
|  | hsa-miR-762 | 100.0 | -0.52 | -0.05 | 1.23 | (1.01, | 1.51) | 0.05 | 0.109 |
|  | hsa-miR-769-3p | 100.0 | -0.46 | -0.05 | 1.31 | (1.07, | 1.59) | 0.01 | 0.076 |
|  | hsa-miR-874 | 100.0 | -0.39 | -0.05 | 1.22 | (1.04, | 1.44) | 0.02 | 0.091 |
|  | hsa-miR-877-5p | 100.0 | -0.18 | 0.16 | 1.29 | (1.07, | 1.56) | 0.01 | 0.076 |
|  | hsa-miR-887 | 99.9 | -0.50 | -0.09 | 1.25 | (1.03, | 1.53) | 0.03 | 0.097 |
|  | hsa-miR-892b | 98.8 | -0.61 | -0.04 | 1.25 | (1.05, | 1.50) | 0.01 | 0.084 |
|  | hsa-miR-939-5p | 100.0 | -0.41 | 0.01 | 1.21 | (1.03, | 1.42) | 0.02 | 0.096 |
|  | hsa-miR-940 | 100.0 | -0.65 | -0.06 | 1.25 | (1.01, | 1.55) | 0.04 | 0.097 |
