# Supplementary material for: Site-specific associations between miRNA expression and survival in colorectal cancer cases
Source: Oncotarget. 2016 Aug 10;7(37):60193–205. doi: 10.18632/oncotarget.11173 (PMC5312378; doi:10.18632/oncotarget.11173)
Supplement: Supplementary file 3 [file oncotarget-07-60193-s003.docx]

| Supplemental Table 4. Differential miRNA expression between carcinoma and normal mucosa associated with survival in rectal cancer cases where FDR q value is ≥0.031 | | | | | | | | | |
| --- | --- | --- | --- | --- | --- | --- | --- | --- | --- |
| miRNA | % expressing | 25th%ile | 75th%ile | HR^1^ | 95% (CI) | | p-value | FDR q-value | |
| hsa-miR-106b-5p | 71.3 | 0.69 | 2.64 | 0.81 | (0.68, | 0.95) | 0.0139 | 0.0335 |  |
| hsa-miR-1202 | 100.0 | -0.36 | 0.10 | 1.20 | (1.04, | 1.38) | 0.0155 | 0.0349 |  |
| hsa-miR-1203 | 64.6 | -1.58 | 0.00 | 0.79 | (0.66, | 0.95) | 0.0127 | 0.0331 |  |
| hsa-miR-1225-3p | 100.0 | -0.47 | 0.05 | 1.18 | (1.02, | 1.35) | 0.0264 | 0.0431 |  |
| hsa-miR-1226-5p | 100.0 | -0.34 | 0.06 | 1.19 | (1.05, | 1.36) | 0.0101 | 0.0311 |  |
| hsa-miR-1227-5p | 100.0 | -0.61 | -0.06 | 1.22 | (1.06, | 1.41) | 0.0131 | 0.0333 |  |
| hsa-miR-1228-3p | 100.0 | -0.34 | 0.12 | 1.17 | (1.03, | 1.34) | 0.0228 | 0.0395 |  |
| hsa-miR-1268b | 100.0 | -0.31 | 0.20 | 1.20 | (1.04, | 1.39) | 0.0182 | 0.0358 |  |
| hsa-miR-1275 | 100.0 | -0.51 | -0.02 | 1.17 | (1.02, | 1.35) | 0.0342 | 0.0514 |  |
| hsa-miR-1291 | 69.1 | 0.00 | 1.44 | 0.87 | (0.76, | 0.99) | 0.0290 | 0.0457 |  |
| hsa-miR-1299 | 100.0 | -0.33 | 0.12 | 1.14 | (1.01, | 1.29) | 0.0332 | 0.0501 |  |
| hsa-miR-1321 | 78.6 | -0.28 | 0.50 | 0.93 | (0.86, | 1.00) | 0.0448 | 0.0606 |  |
| hsa-miR-134 | 100.0 | -0.46 | -0.01 | 1.21 | (1.05, | 1.39) | 0.0104 | 0.0314 |  |
| hsa-miR-1469 | 100.0 | -0.42 | -0.02 | 1.16 | (1.01, | 1.33) | 0.0442 | 0.0603 |  |
| hsa-miR-149-3p | 100.0 | -0.39 | 0.08 | 1.22 | (1.05, | 1.42) | 0.0122 | 0.0328 |  |
| hsa-miR-150-3p | 100.0 | -0.34 | 0.04 | 1.20 | (1.04, | 1.37) | 0.0137 | 0.0335 |  |
| hsa-miR-150-5p | 98.6 | -2.49 | -0.71 | 0.85 | (0.72, | 1.00) | 0.0294 | 0.0459 |  |
| hsa-miR-1587 | 100.0 | -0.42 | 0.09 | 1.17 | (1.02, | 1.35) | 0.0394 | 0.0567 |  |
| hsa-miR-187-5p | 99.4 | -0.60 | -0.05 | 1.11 | (1.01, | 1.22) | 0.0442 | 0.0603 |  |
| hsa-miR-188-5p | 100.0 | -0.49 | -0.03 | 1.16 | (1.01, | 1.33) | 0.0409 | 0.0580 |  |
| hsa-miR-1914-3p | 100.0 | -0.35 | 0.01 | 1.20 | (1.04, | 1.38) | 0.0141 | 0.0337 |  |
| hsa-miR-192-5p | 99.4 | -1.15 | 0.18 | 0.84 | (0.74, | 0.96) | 0.0160 | 0.0349 |  |
| hsa-miR-193a-5p | 99.8 | -0.15 | 0.19 | 1.14 | (1.02, | 1.27) | 0.0171 | 0.0352 |  |
| hsa-miR-194-3p | 99.9 | -0.60 | 0.02 | 0.82 | (0.69, | 0.99) | 0.0385 | 0.0560 |  |
| hsa-miR-196a-5p | 66.9 | -0.42 | 1.91 | 0.82 | (0.69, | 0.97) | 0.0244 | 0.0410 |  |
| hsa-miR-2467-3p | 99.9 | -0.03 | 0.27 | 1.15 | (1.01, | 1.31) | 0.0394 | 0.0567 |  |
| hsa-miR-26a-5p | 99.7 | -0.36 | 0.66 | 0.83 | (0.71, | 0.97) | 0.0160 | 0.0349 |  |
| hsa-miR-2861 | 100.0 | -0.62 | -0.08 | 1.22 | (1.06, | 1.42) | 0.0109 | 0.0320 |  |
| hsa-miR-3138 | 100.0 | -0.31 | 0.04 | 1.19 | (1.03, | 1.36) | 0.0196 | 0.0373 |  |
| hsa-miR-3141 | 100.0 | -0.12 | 0.27 | 1.16 | (1.02, | 1.33) | 0.0290 | 0.0457 |  |
| hsa-miR-3149 | 52.6 | -1.43 | 0.44 | 0.84 | (0.72, | 0.98) | 0.0398 | 0.0567 |  |
| hsa-miR-3150b-5p | 98.2 | -0.75 | -0.06 | 1.15 | (1.02, | 1.29) | 0.0199 | 0.0376 |  |
| hsa-miR-3180-3p | 98.2 | -0.35 | 0.11 | 1.10 | (1.02, | 1.19) | 0.0215 | 0.0390 |  |
| hsa-miR-3185 | 99.9 | -0.28 | 0.18 | 1.19 | (1.03, | 1.38) | 0.0228 | 0.0395 |  |
| hsa-miR-3196 | 100.0 | -0.53 | -0.07 | 1.23 | (1.05, | 1.44) | 0.0124 | 0.0328 |  |
| hsa-miR-3200-5p | 99.9 | -0.47 | -0.05 | 1.22 | (1.04, | 1.43) | 0.0182 | 0.0358 |  |
| hsa-miR-3202 | 99.8 | -0.26 | 0.11 | 1.15 | (1.01, | 1.31) | 0.0430 | 0.0599 |  |
| hsa-miR-320c | 100.0 | -0.50 | 0.14 | 1.17 | (1.01, | 1.35) | 0.0477 | 0.0629 |  |
| hsa-miR-324-3p | 100.0 | 0.06 | 0.48 | 1.20 | (1.03, | 1.38) | 0.0149 | 0.0344 |  |
| hsa-miR-33b-3p | 100.0 | -0.26 | 0.08 | 1.13 | (1.02, | 1.25) | 0.0222 | 0.0392 |  |
| hsa-miR-345-3p | 100.0 | -0.23 | 0.23 | 1.19 | (1.02, | 1.38) | 0.0236 | 0.0403 |  |
| hsa-miR-3610 | 100.0 | -0.57 | -0.01 | 1.22 | (1.04, | 1.42) | 0.0142 | 0.0337 |  |
| hsa-miR-361-5p | 81.6 | 0.25 | 1.55 | 0.88 | (0.77, | 0.99) | 0.0379 | 0.0557 |  |
| hsa-miR-3620-5p | 100.0 | -0.45 | 0.01 | 1.18 | (1.03, | 1.36) | 0.0280 | 0.0448 |  |
| hsa-miR-3622b-5p | 100.0 | -0.18 | 0.20 | 1.22 | (1.03, | 1.44) | 0.0221 | 0.0392 |  |
| hsa-miR-3652 | 100.0 | -0.31 | 0.05 | 1.18 | (1.02, | 1.36) | 0.0301 | 0.0468 |  |
| hsa-miR-365a-3p | 51.2 | 0.00 | 2.25 | 0.83 | (0.70, | 0.99) | 0.0282 | 0.0449 |  |
| hsa-miR-3663-3p | 100.0 | -0.43 | 0.06 | 1.21 | (1.04, | 1.41) | 0.0229 | 0.0395 |  |
| hsa-miR-3665 | 100.0 | -0.56 | -0.01 | 1.23 | (1.05, | 1.43) | 0.0119 | 0.0326 |  |
| hsa-miR-3666 | 99.8 | -0.35 | 0.04 | 1.18 | (1.04, | 1.34) | 0.0175 | 0.0356 |  |
| hsa-miR-371b-5p | 100.0 | -0.78 | -0.13 | 1.21 | (1.04, | 1.40) | 0.0208 | 0.0386 |  |
| hsa-miR-378e | 53.2 | -0.14 | 0.60 | 0.89 | (0.82, | 0.97) | 0.0177 | 0.0357 |  |
| hsa-miR-378f | 96.5 | -0.07 | 0.20 | 1.06 | (1.01, | 1.11) | 0.0271 | 0.0439 |  |
| hsa-miR-3917 | 100.0 | -0.25 | 0.11 | 1.17 | (1.01, | 1.34) | 0.0331 | 0.0501 |  |
| hsa-miR-3937 | 100.0 | -0.36 | 0.08 | 1.20 | (1.04, | 1.38) | 0.0132 | 0.0333 |  |
| hsa-miR-3940-5p | 100.0 | -0.54 | 0.01 | 1.22 | (1.05, | 1.42) | 0.0157 | 0.0349 |  |
| hsa-miR-424-3p | 99.9 | 0.28 | 0.94 | 0.81 | (0.69, | 0.96) | 0.0194 | 0.0373 |  |
| hsa-miR-4270 | 100.0 | -0.34 | 0.11 | 1.20 | (1.05, | 1.37) | 0.0119 | 0.0326 |  |
| hsa-miR-4298 | 100.0 | -0.05 | 0.33 | 1.16 | (1.02, | 1.33) | 0.0230 | 0.0395 |  |
| hsa-miR-4314 | 100.0 | -0.28 | 0.04 | 1.16 | (1.02, | 1.32) | 0.0325 | 0.0500 |  |
| hsa-miR-4323 | 99.5 | -1.02 | -0.37 | 1.19 | (1.03, | 1.37) | 0.0161 | 0.0349 |  |
| hsa-miR-4327 | 100.0 | -0.20 | 0.21 | 1.19 | (1.04, | 1.37) | 0.0155 | 0.0349 |  |
| hsa-miR-432-5p | 54.3 | -0.18 | 0.69 | 0.89 | (0.81, | 0.97) | 0.0137 | 0.0335 |  |
| hsa-miR-4429 | 100.0 | -0.26 | 0.13 | 1.18 | (1.04, | 1.34) | 0.0164 | 0.0352 |  |
| hsa-miR-4433-3p | 100.0 | -0.46 | 0.13 | 1.20 | (1.04, | 1.39) | 0.0167 | 0.0352 |  |
| hsa-miR-4436a | 73.9 | -0.22 | 0.53 | 0.94 | (0.88, | 1.00) | 0.0412 | 0.0581 |  |
| hsa-miR-4442 | 100.0 | -0.21 | 0.09 | 1.14 | (1.01, | 1.29) | 0.0397 | 0.0567 |  |
| hsa-miR-4459 | 100.0 | -0.64 | -0.09 | 1.22 | (1.05, | 1.41) | 0.0128 | 0.0331 |  |
| hsa-miR-4463 | 100.0 | -0.31 | 0.11 | 1.20 | (1.05, | 1.38) | 0.0128 | 0.0331 |  |
| hsa-miR-4466 | 100.0 | -0.52 | 0.01 | 1.23 | (1.06, | 1.42) | 0.0104 | 0.0314 |  |
| hsa-miR-4476 | 100.0 | -0.31 | 0.03 | 1.14 | (1.01, | 1.29) | 0.0440 | 0.0603 |  |
| hsa-miR-4481 | 100.0 | -0.29 | 0.07 | 1.16 | (1.03, | 1.31) | 0.0207 | 0.0386 |  |
| hsa-miR-4508 | 100.0 | -0.54 | -0.09 | 1.21 | (1.04, | 1.40) | 0.0168 | 0.0352 |  |
| hsa-miR-4526 | 78.4 | -0.31 | 0.15 | 0.95 | (0.90, | 1.00) | 0.0326 | 0.0500 |  |
| hsa-miR-4638-5p | 57.8 | -0.80 | 0.13 | 0.91 | (0.84, | 1.00) | 0.0460 | 0.0612 |  |
| hsa-miR-4654 | 51.1 | -0.04 | 0.84 | 0.89 | (0.82, | 0.98) | 0.0185 | 0.0360 |  |
| hsa-miR-4655-5p | 100.0 | -0.23 | 0.15 | 1.16 | (1.01, | 1.33) | 0.0328 | 0.0500 |  |
| hsa-miR-4664-3p | 100.0 | -0.52 | -0.06 | 1.20 | (1.03, | 1.39) | 0.0221 | 0.0392 |  |
| hsa-miR-4665-3p | 100.0 | -0.58 | 0.03 | 1.19 | (1.02, | 1.39) | 0.0273 | 0.0439 |  |
| hsa-miR-4665-5p | 100.0 | -0.28 | 0.10 | 1.20 | (1.04, | 1.38) | 0.0149 | 0.0344 |  |
| hsa-miR-4685-5p | 100.0 | -0.22 | 0.12 | 1.20 | (1.04, | 1.38) | 0.0137 | 0.0335 |  |
| hsa-miR-4690-5p | 100.0 | -0.65 | -0.15 | 1.20 | (1.04, | 1.39) | 0.0172 | 0.0352 |  |
| hsa-miR-4707-5p | 100.0 | -0.48 | 0.16 | 1.20 | (1.02, | 1.41) | 0.0327 | 0.0500 |  |
| hsa-miR-4710 | 100.0 | -0.15 | 0.24 | 1.17 | (1.01, | 1.36) | 0.0422 | 0.0593 |  |
| hsa-miR-4725-3p | 98.4 | -0.41 | 0.06 | 1.12 | (1.02, | 1.22) | 0.0226 | 0.0395 |  |
| hsa-miR-4734 | 100.0 | -0.49 | 0.00 | 1.20 | (1.03, | 1.39) | 0.0239 | 0.0404 |  |
| hsa-miR-4741 | 100.0 | -0.37 | 0.15 | 1.21 | (1.04, | 1.41) | 0.0247 | 0.0411 |  |
| hsa-miR-4749-3p | 99.0 | -0.93 | -0.34 | 1.16 | (1.02, | 1.33) | 0.0257 | 0.0424 |  |
| hsa-miR-4749-5p | 100.0 | -0.16 | 0.13 | 1.16 | (1.02, | 1.33) | 0.0272 | 0.0439 |  |
| hsa-miR-4763-3p | 100.0 | -0.43 | 0.09 | 1.19 | (1.02, | 1.39) | 0.0358 | 0.0533 |  |
| hsa-miR-4767 | 100.0 | -0.39 | 0.10 | 1.16 | (1.01, | 1.34) | 0.0448 | 0.0606 |  |
| hsa-miR-4783-3p | 100.0 | -0.46 | 0.01 | 1.25 | (1.06, | 1.48) | 0.0130 | 0.0333 |  |
| hsa-miR-4785 | 60.8 | -0.92 | 0.56 | 0.86 | (0.74, | 0.99) | 0.0437 | 0.0603 |  |
| hsa-miR-4793-5p | 100.0 | -0.15 | 0.19 | 1.16 | (1.01, | 1.34) | 0.0435 | 0.0603 |  |
| hsa-miR-493-3p | 73.9 | -0.62 | 0.76 | 0.81 | (0.69, | 0.95) | 0.0156 | 0.0349 |  |
| hsa-miR-498 | 100.0 | -0.39 | 0.04 | 1.18 | (1.04, | 1.35) | 0.0105 | 0.0314 |  |
| hsa-miR-501-3p | 84.8 | 0.56 | 1.73 | 0.82 | (0.70, | 0.95) | 0.0160 | 0.0349 |  |
| hsa-miR-5088 | 100.0 | -0.46 | 0.00 | 1.23 | (1.04, | 1.44) | 0.0165 | 0.0352 |  |
| hsa-miR-5093 | 74.0 | -0.49 | 0.27 | 0.91 | (0.84, | 0.99) | 0.0210 | 0.0386 |  |
| hsa-miR-5100 | 100.0 | -0.70 | 0.01 | 1.25 | (1.05, | 1.48) | 0.0116 | 0.0326 |  |
| hsa-miR-5194 | 100.0 | -0.40 | 0.02 | 1.18 | (1.03, | 1.35) | 0.0183 | 0.0358 |  |
| hsa-miR-5195-3p | 100.0 | -0.38 | 0.08 | 1.20 | (1.04, | 1.38) | 0.0181 | 0.0358 |  |
| hsa-miR-5195-5p | 85.9 | -0.13 | 0.57 | 0.92 | (0.86, | 0.99) | 0.0213 | 0.0389 |  |
| hsa-miR-519e-5p | 54.4 | -0.26 | 0.14 | 0.95 | (0.91, | 0.99) | 0.0217 | 0.0390 |  |
| hsa-miR-548q | 100.0 | -0.77 | -0.17 | 1.22 | (1.05, | 1.43) | 0.0146 | 0.0344 |  |
| hsa-miR-5580-3p | 98.0 | -0.16 | 0.12 | 1.07 | (1.01, | 1.14) | 0.0250 | 0.0414 |  |
| hsa-miR-5703 | 100.0 | -0.70 | -0.02 | 1.18 | (1.03, | 1.35) | 0.0178 | 0.0357 |  |
| hsa-miR-5739 | 100.0 | -0.29 | 0.11 | 1.17 | (1.02, | 1.35) | 0.0363 | 0.0538 |  |
| hsa-miR-575 | 100.0 | -0.39 | 0.04 | 1.21 | (1.04, | 1.40) | 0.0160 | 0.0349 |  |
| hsa-miR-584-5p | 100.0 | -0.30 | 0.04 | 1.17 | (1.03, | 1.32) | 0.0120 | 0.0326 |  |
| hsa-miR-601 | 100.0 | -0.36 | 0.01 | 1.19 | (1.05, | 1.34) | 0.0106 | 0.0314 |  |
| hsa-miR-6069 | 100.0 | -0.33 | 0.19 | 1.17 | (1.03, | 1.34) | 0.0170 | 0.0352 |  |
| hsa-miR-6074 | 99.2 | -0.18 | 0.14 | 1.10 | (1.03, | 1.18) | 0.0119 | 0.0326 |  |
| hsa-miR-6075 | 100.0 | -0.61 | -0.09 | 1.18 | (1.02, | 1.37) | 0.0368 | 0.0543 |  |
| hsa-miR-6084 | 99.5 | -0.38 | 0.15 | 1.17 | (1.03, | 1.32) | 0.0210 | 0.0386 |  |
| hsa-miR-6085 | 100.0 | -0.30 | 0.08 | 1.14 | (1.00, | 1.30) | 0.0195 | 0.0373 |  |
| hsa-miR-6086 | 100.0 | -0.41 | 0.04 | 1.20 | (1.04, | 1.38) | 0.0171 | 0.0352 |  |
| hsa-miR-6132 | 100.0 | -0.34 | 0.11 | 1.18 | (1.02, | 1.37) | 0.0264 | 0.0431 |  |
| hsa-miR-6165 | 100.0 | -0.07 | 0.35 | 1.17 | (1.03, | 1.34) | 0.0216 | 0.0390 |  |
| hsa-miR-630 | 100.0 | -0.72 | -0.06 | 1.21 | (1.05, | 1.39) | 0.0110 | 0.0320 |  |
| hsa-miR-6500-3p | 60.4 | -0.72 | 0.41 | 0.89 | (0.80, | 0.98) | 0.0245 | 0.0410 |  |
| hsa-miR-6512-5p | 100.0 | -0.37 | 0.05 | 1.19 | (1.04, | 1.36) | 0.0139 | 0.0335 |  |
| hsa-miR-652-5p | 100.0 | -0.34 | 0.04 | 1.15 | (1.01, | 1.32) | 0.0457 | 0.0610 |  |
| hsa-miR-6723-5p | 100.0 | -0.31 | 0.07 | 1.16 | (1.01, | 1.34) | 0.0471 | 0.0624 |  |
| hsa-miR-760 | 100.0 | -0.23 | 0.16 | 1.15 | (1.02, | 1.31) | 0.0292 | 0.0458 |  |
| hsa-miR-762 | 100.0 | -0.56 | -0.08 | 1.21 | (1.04, | 1.41) | 0.0166 | 0.0352 |  |
| hsa-miR-887 | 100.0 | -0.52 | -0.12 | 1.21 | (1.04, | 1.40) | 0.0148 | 0.0344 |  |
| hsa-miR-93-5p | 94.6 | 0.87 | 1.98 | 0.87 | (0.77, | 0.97) | 0.0194 | 0.0373 |  |
| hsa-miR-940 | 100.0 | -0.66 | -0.06 | 1.21 | (1.03, | 1.42) | 0.0206 | 0.0386 |  |
| ^1^Hazard Ratios (HR) and 95% Confidence Intervals (CI) adjusted for age, sex, AJCC stage, and MSI tumor status | | | | | | | | | |
